# Implications of ammonia stress for the pathogenicity of *Shewanella* spp. in *Oreochromis niloticus*: effects on hematological, biochemical, immunological, and histopathological parameters

**DOI:** 10.1186/s12917-024-04175-9

**Published:** 2024-07-18

**Authors:** Rasha M. Reda, Abdelhakeem El-Murr, Nehal A. Abdel-Basset, Mohamed M. M. Metwally, Rowida E. Ibrahim

**Affiliations:** 1 Department of Aquatic Animal Medicine, Faculty of Veterinary Medicine, Zagazig University, PO Box 44511, Zagazig, Sharkia Egypt; 2 Department of Pathology, Faculty of Veterinary Medicine, Zagazig University, PO Box 44511, Zagazig, Sharkia Egypt; 3 Department of Pathology and Clinical Pathology, Faculty of Veterinary Medicine, King Salman International University, Ras Sudr, Egypt

**Keywords:** *Shewanella*, Nile tilapia, Ammonia stress, Cortisol, Immunity

## Abstract

Environmental stressors (such as ammonia) in aquaculture could increase the risk of pathogenicity, posing a more severe threat to farmed fish. The aim of this study was to investigate the effects of ammonia stress on the pathogenicity of *Shewanella* spp. in *Oreochromis niloticus*. First, a 96-hour static test was used to determine the median lethal concentration (LC_50_) of unionized ammonia to Nile tilapia. After 96 h of exposure, the Un-ionized ammonia (UIA) LC_50_ was estimated to be 4.26 mg/L. Second, an experiment was conducted to test the effect of unionized ammonia stress on the pathogenicity of *Shewanella* spp. in *O. niloticus* for 30 days. A study involved 180 fish divided into six groups, with the first group serving as a control. The second group (AMN1/10) and the third group (AMN1/20) were not challenged and were exposed to 1/10 (0.42 mg/L) and 1/20 (0.21 mg/L) of the 96-hour LC_50_ of UIA, respectively. Then 0.2 mL (0.14 × 10^5^) of *Shewanella* spp. was intraperitoneally injected into the fourth (SH), fifth (SH + AMN1/10), and sixth (SH + AMN1/20) groups, which were subjected to 0, 1/10 (0.42 mg/L), and 1/20 (0.21 mg/L) of the 96-hour LC_50_ of UIA, respectively. The survival rate, hematological indices, immunological parameters, and antioxidant activity of the fish significantly decreased when they were exposed to ammonia and *Shewanella* infection separately or together. Histopathological changes were also observed in the kidney and liver. Furthermore, both individual and combined exposures significantly altered renal and hepatic function, with notable increases in glucose and cortisol levels, as well as in the expression of proinflammatory cytokine genes (*TNF-α* and *IL-1ß*). However, the detrimental effects of co-exposure to ammonia stress and *Shewanella* infection were greater than those of separate exposures. As a result, we may say that increased ammonia concentrations enhance the infection of *Shewanella* spp. These findings could contribute to a better understanding of *Shewanella* infection in Nile tilapia.

## Introduction

In a world where environmental issues and growing populations are major concerns, the pursuit of sustainable development is more important than ever. In this effort, aquaculture is seen as a potent tool that provides a wide range of answers to complex problems that Sustainable Development Goals (SDGs) aim to solve [1, 2]. SDG 2, “Zero Hunger,” aims to end hunger and malnutrition by 2030. Aquaculture stands as a potent weapon in this fight. It provides a reliable and nutritious source of protein that is essential for healthy growth and development, particularly in vulnerable populations [1, 3, 4]. Compared to terrestrial livestock, aquaculture offers higher protein conversion rates, requiring less feed and land per unit of protein produced [5, 6]. This makes it a particularly efficient option for food production in resource-constrained environments.

Despite its remarkable potential, aquaculture faces challenges, such as disease outbreaks and environmental stressors, that need to be addressed for its sustainable development. Ammonia, a byproduct of fish metabolism, is a natural component of aquatic environments. However, at high concentrations, it becomes highly toxic to aquatic organisms. In aquaculture, where stocking densities are often high, ammonia levels can easily rise above tolerable limits due to factors such as overfeeding, poor water quality management, and inadequate biofiltration [7–9]. This results in a cascade of negative consequences for farmed fish species [10–12]. One of the most concerning consequences of ammonia stress is its immunosuppressive effect. Elevated ammonia levels impair the immune function of fish and shellfish, reducing their ability to fight infections and resist disease. This weakens their defense against opportunistic pathogens, paving the way for disease outbreaks, especially through opportunistic pathogens [13–16]. In addition, disruptions in the microbiome can occur, allowing previously harmless or suppressed microbes to proliferate or introduce completely new microbes from the environment [14, 17, 18].

*Shewanella* is a genus of rod-shaped, gram-negative bacteria belonging to the Shewanellaceae family of the order Alteromonadales [19]. While often overshadowed by other aquatic pathogens, *Shewanella* bacteria can emerge as opportunistic threats in aquaculture settings [20]. These adaptable microorganisms, commonly found in diverse aquatic environments, pose a risk to farmed fish when stressed or weakened by factors such as ammonia stress [14, 18, 21]. *Shewanella* infection manifests in various ways depending on the species and strain involved. Skin lesions, hemorrhages in internal organs, and large-scale mortality were recorded in different cases, leading to severe economic losses [22–24].

The rise of *Shewanella* in aquaculture poses a new challenge to the industry. Previously, this bacterium has not been a major cause for concern compared to other pathogenic bacteria such as *Aeromonas*, *Streptococcus*, and *Edwardsiella*. However, recent reports suggest an increase in *Shewanella*-related infections and mortality rates within fish farms [22, 24–26]. To the best of our knowledge, no study has looked at how *Shewanella* spp. infections change with various concentrations of ammonia in *Oreochromis niloticus* (*O. niloticus*). Therefore, the goal of this study was to investigate how ammonia stress, which is considered a natural stressor in aquaculture systems, affects the pathogenicity of *Shewanella* spp. in *O. niloticus*.

## Materials and methods

### Fish

Three hundred and sixty *O. niloticus*, with an initial average weight of 26.13 ± 0.07 g, were acquired from the Fish Research Unit of the Faculty of Veterinary Medicine at Zagazig University in Egypt. A total of 180 fish were used to determine the median 96-hour lethal concentration (96-h LC_50_) of unionized ammonia (UIA), and the remaining 180 fish were used for the experimental trial. Fish were carefully transported to the Department of Aquatic Animal Medicine lab located on the same faculty. To help the fish adapt to laboratory conditions, they were placed in 100-liter aquariums with constant aeration and dechlorinated tap water for a period of 14 days. Fish were inspected for health upon arrival before being used in the experiment [27, 28]. According to APHA [29], the water parameters were adjusted during both the acclimatization and experimental phases. The water parameters were measured as follows: pH 6.60 ± 0.50, temperature 22.5 ± 0.50 ℃, ammonia 0.02 ± 0.001 mg/L, nitrite 0.02 ± 0.012 mg/L, nitrate 0.15 ± 0.02 mg/L, dissolved oxygen 5.50 ± 0.40 mg/L, and 12-hour photoperiod: 12-hour darkness. During the acclimatization and experimental period, The amount of food provided in each aquarium was calculated to equal 3% of the total live fish weight. Fish were fed a control diet (Table 1) [30] designed to fulfill the standard nutritional needs of tilapia. Fish were fed a pelleted diet (1.5 mm) at 9:00 a.m. and 3:00 p.m. Pellets were evenly distributed across the water surface of the aquarium. Feeding continued until fish showed signs of satiety, such as spitting out pellets, or until no more pellets were accepted by fish [31, 32].Every day, the old water was replaced with fresh, dechlorinated water.

**Table 1:** Composition of basal diet (%)

Ingredients	Percentage of ingredient	
Fish meal (65.4% CP)	40	
Soybean meal (44%)	20	
Yellow corn	13	
Wheat flour	15	
Wheat Bran	2	
Fish oil	7	
Monocalcium phosphate	2	
^(1)^ Vitamin mixture	0.45	
^(2)^ Mineral mixture	0.55	
**Chemical analyses (% DM)**		
Crud Protein	38.90	
Crude fat	10.50	
Ash	5.84	

^(1)^**Vitamin mix (IU or mg kg diet)**: vitamin A, 16,000 IU; vitamin D, 8000 IU; vitamin K, 14.72; thiamin, 17.8; riboflavin, 48; pyridoxine, 29.52; cynocobalamine, 0.24, tocopherols acetate, 160; ascorbic acid (35%), 800; niacinamide, 79.2; calcium-D- pantothenate,73.6; folic acid, 6.4; biotin, 0.64 L-carnitine, 100

^(2)^**Mineral mix (mg kg diet)**: Cu (CuSO4), 2.0; Zn (ZnSO4), 34.4; Mn (MnSO4), 6.2; Fe (FeSO4), 21.1; I (Ca (IO3)2), 1.63; Se (Na2SeO3), 0.18; Co (CoCl2), 0.24; Mg (MgSO4.H2O), 52.7

### Ammonium chloride

The source of ammonia in this study was ammonium chloride (NH_4_Cl), which was purchased from El-Gomhouria Co., Egypt. Daily, an NH_4_Cl solution was prepared by the combination of NH_4_Cl and dechlorinated, filtered potable water.

### Shewanella spp. isolate

The *Shewanella* spp. (GenBank accession number: OP942237) used in this study were previously isolated from *O. niloticus* that were moribund. *Shewanella* spp. identification, characterization, and determination of their median lethal dose (LD_50_) were determined as mentioned in Reda, El-Murr [33]. The *Shewanella* spp. isolate was re-cultured for 24–48 h at 22 °C on tryptic soy agar medium (Oxoid, England). The bacterial isolate concentration was adjusted to 0.14 × 10^5^ CFU/mL [33] using McFarland Standard No. 0.5 for the experimental challenge.

### Determination of the median 96-hour lethal concentration (96-h LC_50_) of unionized ammonia (UIA)

**Fig. 1:**
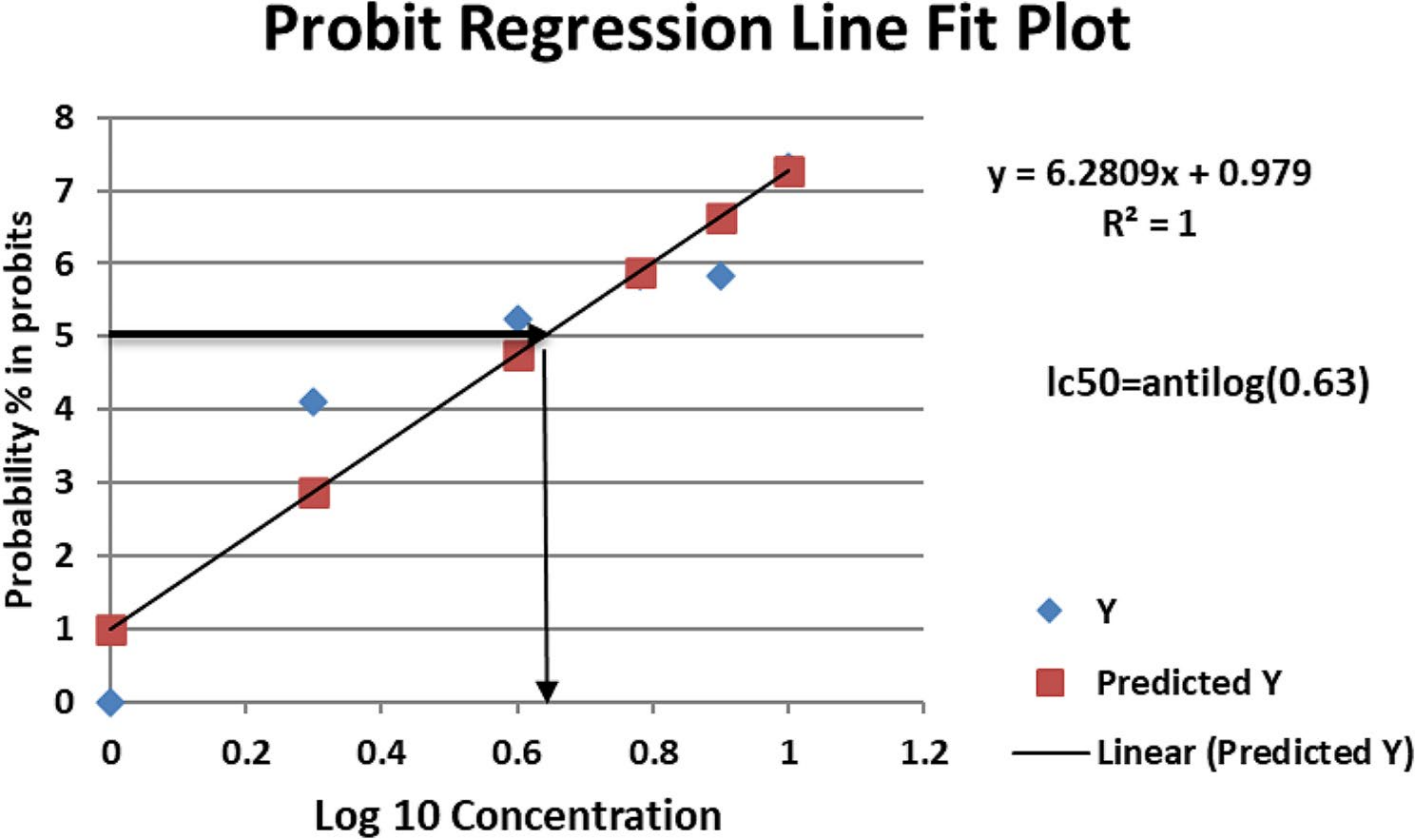
Probit analysis graph showing LC_50_ of unionized ammonia in *Oreochromis niloticus*

One hundred eighty fish were divided into six groups. Each group had three replicates (10 fish per replicate, 30 fish per group). The groups from one to six were exposed to 0, 2, 4, 6, 8, or 10 mg/L of UIA. The fish were subjected to the previously specified UIA concentrations in a semi-static condition, with daily UIA measurements and water renewals (if necessary) to maintain a constant ammonia concentration [34, 35]. During the 96-hour exposure period, the fish were not fed in accordance with OECD recommendations [36]. Fish mortality was recorded daily and removed immediately. The 96-h LC_50_ value was calculated using probit analysis with IBM^®^ SPSS^®^ Statistics 23 [37]. After 96 h of exposure, the UIA LC_50_ was estimated to be 4.26 mg/L (Fig. 1).

### Experimental plan

For 30 days, 180 fish were divided into six groups in triplicate (30 fish per group; 10 fish per replicate). The first group served as the control group (CONT), the second group (AMN1/10), and the third group (AMN1/20) were exposed to 1/10 (0.42 mg/L) and 1/20 (0.21 mg/L) of the 96-hour LC_50_ of UIA, respectively, without being challenged. The fourth (SH), fifth (SH + AMN1/10), and sixth (SH + AMN1/20) groups were anesthetized with a 100 mg/L benzocaine solution [38] and then intraperitoneal (IP) injected with 0.2 mL (0.14 × 10^5^) of *Shewanella* spp. and exposed to 0, 1/10 (0.42 mg/L), or 1/20 (0.21 mg/L) of the 96-h LC_50_ of UIA, respectively. Water was completely changed daily with suction to uneaten food and waste materials and replaced with dechlorinated tap water with adjustment of the UIA concentration for the UIA-exposed group. The actual UIA concentration was measured twice a day for 24 h using the HI-715 (HANNA, Milan, Italy) before and after water exchange and the addition of a freshly prepared ammonium chloride solution to maintain UIA at the levels that were wanted [39]. UIA levels before and after water exchange were 0.50 ± 0.51 and 0.42 ± 0.30 mg/L, respectively (*n* = 9/treatment), for AMN1/10 and AMN1/10 + SH, and 0.32 ± 0.30 and 0.21 ± 0.25 mg/L, respectively (*n* = 9/treatment) for AMN1/20 and AMN1/20 + SH. Throughout the experiment, the clinical signs and mortality of the fish were documented daily.

### Sampling

At the end of the experiment (after 30 days), nine fish per group were chosen at random and anesthetized with a 100 mg/L benzocaine solution [38]. Two sets of blood samples were collected from caudal vessels. To measure phagocytic activity (PA) and hematological indices, one set was taken using syringes that had been heparinized to hold one milliliter of blood. To separate the serum, another set of blood samples was drawn without the use of an anticoagulant and centrifuged for 10 min at 4 °C and 3000 rpm. Until the biochemical and immune parameters were assessed, the serum was kept at -20℃. Liver samples (9 fish/group) from the same fish that were killed by an overdose of benzocaine solution (400 mg/L) were obtained to calculate the oxidant/antioxidant indices [40].

### Analytical parameters

#### Hematological indices

A hemocytometer (Hospitex Diagnostics, Sesto Fiorentino, Italy) was used to count the total number of white blood cells (WBCs) and red blood cells (RBCs). The Jain [41] method was used to measure the packed cell volume (PCV%) and hemoglobin (Hb) content.

#### Biochemical indices

The serum alanine aminotransferase (ALT, Catalog No.; MBS038444), aspartate aminotransferase (AST, Catalogue No.; MBS1601734), and urea (Catalog No.; MBS8305389) levels were determined in accordance with the manufacturer’s guidelines (MyBioSource, Inc., San Diego, CA 92,195 − 3308: USA). The method of Fossati, Prencipe [42] was used to measure the creatinine concentration. Calorimetric analysis was used to measure the serum cortisol and glucose levels using the methods of Saliu, Oluberu [43] and Trinder [44], respectively.

#### Oxidant/antioxidant assays

Using a spectrophotometric method, the oxidant/antioxidant indices in the hepatic homogenate were determined. The protocol described by Reda, Helmy [45] was followed for preparation of the hepatic homogenate. Spectrophotometry was used to estimate the total antioxidant capacity (TAC) (Catalog no. MBS2540515), catalase (CAT) (Catalog no. MBS038818), and malondialdehyde (MDA) (Catalog no. MBS007853) (MyBioSource, Inc., San Diego, CA 92,195 − 3308: USA). Colorimetric measurement of reduced glutathione (GSH) was conducted using the Beutler, Duron [46] method.

#### Immune indices assay

Using heat-inactivated *Candida albicans*, the phagocytic index (PI) and activity (PA%) were calculated using the following formulas [47]:

PI = total no. of phagocytized yeast/no. of phagocytic cells phagocytizing yeast

PA% = (No. of phagocytic cells phagocytizing yeast/Total no. of phagocytic cells counted) × 100

Using spectrophotometry based on the lysis of freeze-dried *Micrococcus lysodeikticus* particles, serum lysozyme (LYZ) activity was determined [48]. Using the method described by Montgomery and Dymock [49], nitric oxide (NO) was measured. Interleukin-1β (IL-1β) (Catalog no. MBS283380) and tumor necrosis factor-α (TNF-α) (Catalog no. MBS704369) were estimated in accordance with the manufacturer’s instructions (MyBioSource, Inc., San Diego, CA 92,195 − 3308: USA).

#### Histopathological study

Liver and kidney specimens (9 fish/group) were sampled and immersed in a 10% neutral buffered formalin fixative for 48 h. The fixed specimens were manually processed by the paraffin technique, sectioned at 4 μm thickness where two Sect. (50 μm apart) per organ per fish were obtained, and the tissue samples were routinely stained with hematoxylin and eosin as described previously by Suvarna, Layton [50]. Next, a multiparametric numeric assessment of the histological condition of the hepatic and renal tissues was performed to determine the organ histological indices was carried out as established by Bernet, Schmidt [51] using an AmScope CMOS C-Mount microscope digital camera (United Scope LLC., CA., USA) attached to a Nikon light microscope (Nikon Inc., NY, USA).

### Statistical analysis

To ensure that the gathered data were regularly distributed, Shapiro-Wilk’s test was used. One-way analysis of variance (ANOVA), was used to identify noteworthy differences between the treatments. To identify differences between means, Tukey multiple range tests were used. Kaplan Meier analysis revealed noteworthy variations in the survival curve. SPSS Version 23 for Windows was used to conduct the statistical analysis (SPSS Inc., Chicago, IL, USA). The 96-h LC_50_ of UIA was determined using probit analysis with IBM^®^ SPSS^®^ Statistics 23. The means ± standard errors (SE) are displayed for the data.

## Results

### Clinical symptoms

**Fig. 2:**
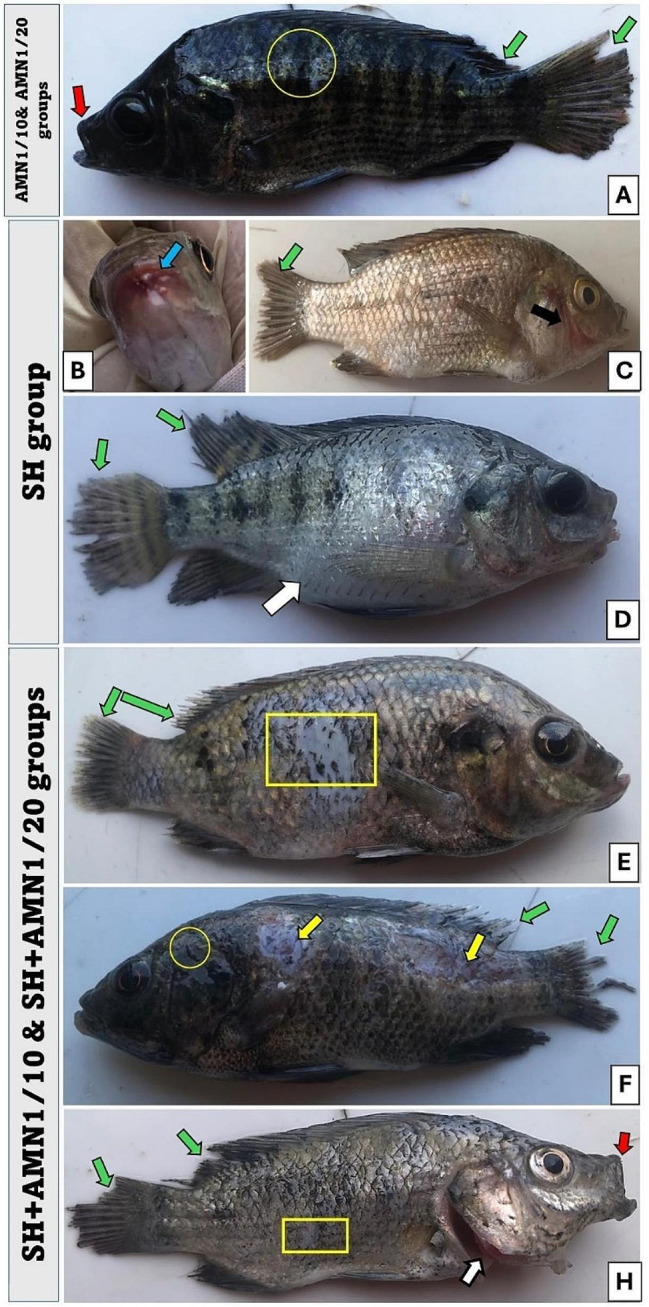
Clinical signs of Nile tilapia challenged with *Shewanella spp.* and reared under different ammonia levels. (**A**) The symptoms in the AMN1/10 and AMN1/20 groups are nearly identical, with an increase in intensity in the AMN1/10 group. The symptoms manifest as darkness in skin coloration (yellow circle), fin rot (green arrows), and an open mouth (red arrow). (**B**,** C**), and (**D**) The SH group manifested hemorrhage in the lower jaw (blue arrow), hemorrhage at the gill cover (black arrow), severe fin rot (green arrow), and ascites in some cases (white arrow). (**E**,** F**), and (**H**) The symptoms in the SH + AMN1/10 and SH + AMN1/20 groups are almost identical, with an increase in intensity in the SH + AMN1/10 group. Fish manifested fin rot (green arrows) and scale loss (yellow rectangular), increased mucus secretion (yellow circle), ulcers in some cases (yellow arrow), an enlarged and opened gill cover (white arrow), and an open mouth (red arrow). AMN1/10, and AMN1/20 groups were non-challenged groups and exposed to 0.42, and 0.21 mg/L of unionized ammonia, respectively. SH, SH + AMN1/10, and SH + AMN1/20 groups were intraperitoneal challenged with Shewanella spp. and exposed to 0, 0.42, and 0.21 mg /L of unionized ammonia, respectively

**Fig. 3:**
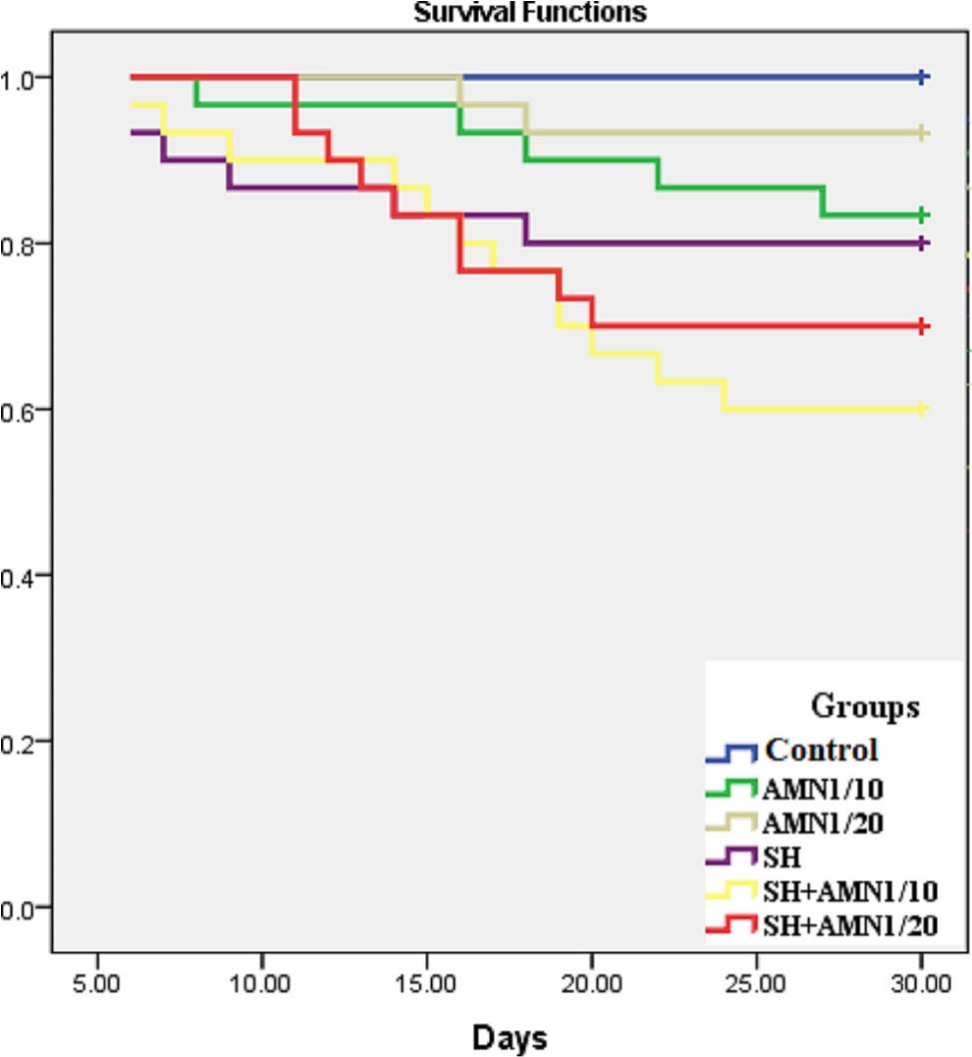
Survival Kaplan Meier curves for Nile tilapia challenged with Shewanella spp. and reared under different ammonia levels. Control, AMN1/10, and AMN1/20 groups were non-challenged groups and exposed to 0, 0.42, and 0.21 mg/L of unionized ammonia, respectively. SH, SH + AMN1/10, and SH + AMN1/20 groups were intraperitoneal challenged with Shewanella spp. and exposed to 0, 0.42, and 0.21 mg/L of ammonium chloride, respectively

**Fig. 4:**
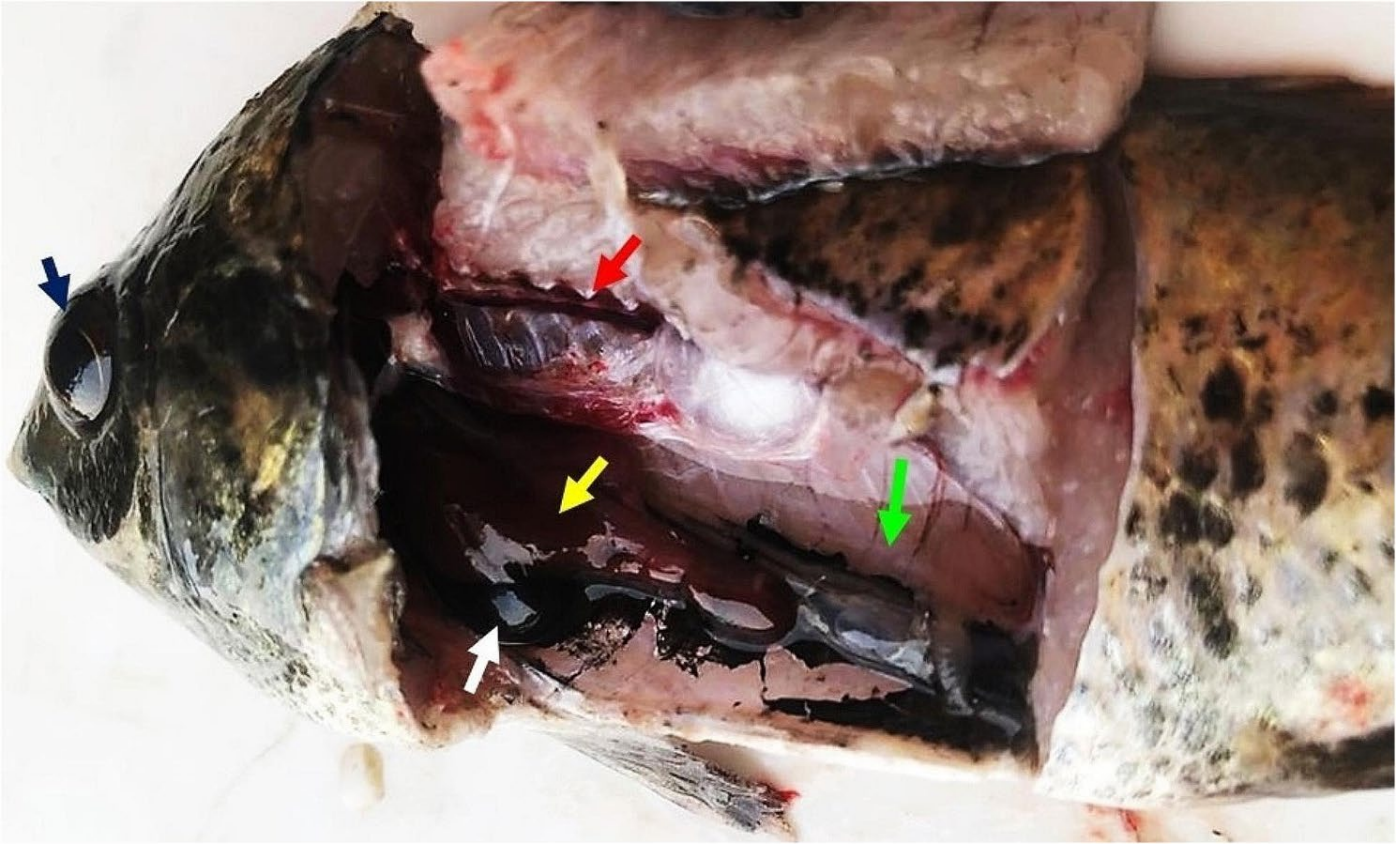
Postmortem findings of Nile tilapia challenged with Shewanella spp. and reared under different ammonia levels showing congested liver (yellow arrow) and kidney (red arrow), enlarged gall bladder (white arrow) with ascitic fluid (green arrow), and slightly protruded eye (blue arrow)

There were no abnormal symptoms recorded in the control group. The symptoms observed in both the AMN1/10 and AMN1/20 groups were almost identical, but they were more severe in the AMN1/10 group. The symptoms presented as darkness of the skin coloration and fin destruction (Fig. 2A). Some of the fish were aggregated near the water surface with lethargy movement and then died with open-mouthed. In the SH group severe fin rot, hemorrhage in the lower jaw was observed in some cases, and hemorrhage at the gill cover and ascites were observed in others (Fig. 2B, C, and D). The symptoms observed in the SH + AMN1/10 and SH + AMN1/20 groups were nearly the same, but they were more severe in the SH + AMN1/10 group (Fig. 2E, F, and H). The fish exhibited symptoms such as fin rot and scale loss, heightened mucus secretion, the presence of ulcers in certain cases, an expanded and exposed gill cover, and an open mouth. According to the Kaplan Meier curve data, 100% of the fish in the control group survived, while 83.3%, 93.3%, 80.0%, 70.0%, and 60.0% of the fish in the AMN1/10, AMN1/20, SH, SH + AMN1/20, and SH + AMN1/10 groups, respectively survived (Fig. 3). Internally, the main lesions recorded in the AMN1/10, and AMN1/20 groups were at the gills. The fish gills exhibited excessive mucus secretion and swelling in the gill filaments. In the AMN1/10 group, some fish displayed severe congestion in the gills, while others had pale gills. The same lesions were nearly the same in the SH, SH + AMN1/10, and SH + AMN1/20 groups, in addition to congestion in the liver, spleen, and kidney, as well as in the ascitic fluid in some fish (Fig. 4).

### Hematological indices

As shown in Table 2, there were no significant differences in the RBC, Hb, PCV%, and WBC counts between the control, AMN1/10, or AMN1/20 groups. The RBC, Hb, PCV%, and WBC counts were significantly lower (*P* = 0.002, *P* = 0.001, *P* = 0.003, *and P* = 0.0001, respectively) in the SH group than in the control group, followed by the SH + AMN1/10 group and the SH + AMN1/20 group.

**Table 2:** Hematological parameters of *O. niloticus* infected with *Shewanella spp* and exposed to ammonia

Groups	RBCs (10^6^/cmm)	Hb (g/dL)	PCV (%)	WBCs (×10^3^/cmm)
CONT	2.11±0.13^a^	4.35±0.33^a^	15.83±1.03^a^	32.63±1.10^a^
AMN1/10	2.15±0.06^a^	4.46±0.23^a^	15.64±0.59^a^	30.50±0.88^a^
AMN1/20	2.22±0.09^a^	4.33±0.21^a^	16.10±0.55^a^	31.33±0.69^a^
SH	1.26±0.33^d^	3.05±0.81^c^	9.18±2.07^c^	18.00±0.70^d^
SH+AMN1/10	1.37±0.14^c^	3.32±0.41^b^	11.03±1.25^b^	21.33±1.44^c^
SH+AMN1/20	1.50±0.16^b^	3.52±0.37^b^	11.16±1.02^b^	27.10±1.74^b^
*P*-value	<0.01	<0.01	<0.01	<0.001

RBCs, total counts of erythrocytes; WBCs, leukocytes; Hb, Hemoglobin, PCV %, Packed Cell Volume. Values (mean ± *SE*) that don’t share superscripts in the same row significantly differ at *P* < 0.05 (One-way ANOVA; Duncan’s post hoc test) (*n*=9/group). CONT, AMN1/10, and AMN1/20 groups were non-challenged groups and exposed to 0, 0.42, and 0.21 mg L^-1^ of ammonium chloride, respectively. SH, SH+AMN1/10, and SH+AMN1/20 groups were intraperitoneal challenged with *Shewanella spp* and exposed to 0, 0.42, and 0.21 mg L^-1^ of ammonium chloride, respectively

### Biochemical indices

**Fig. 5:**
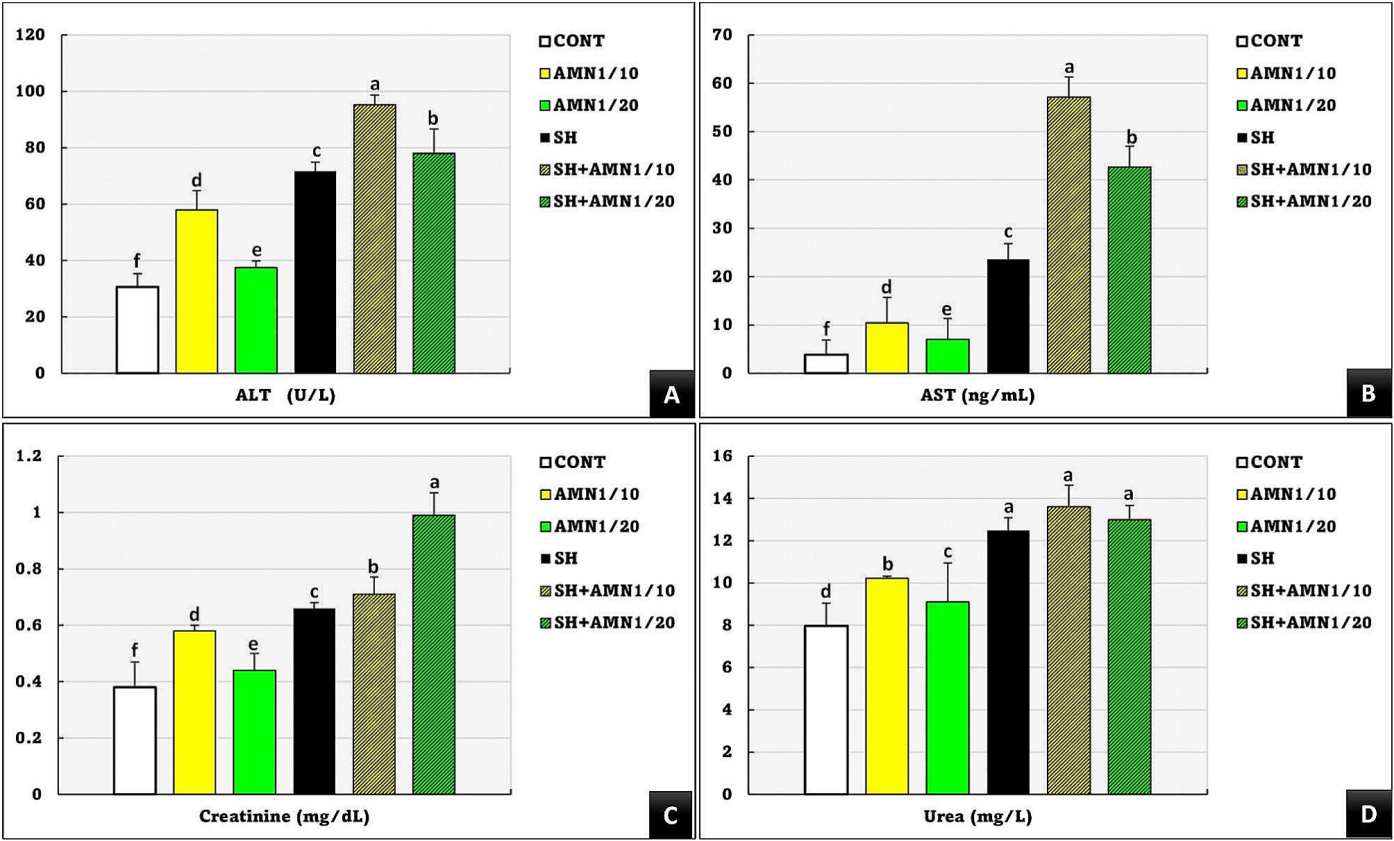
Alanine aminotransferase (ALT, **A**), aspartate transaminase (AST, **B**), creatinine (**C**), and urea (**D**) for Nile tilapia challenged with Shewanella spp. and reared under different ammonia levels. Values are presented as the mean ± SE (*n* = 9/group). The bars with different superscripts (**a**, **b**, **c**, **d**, **e**, and **f**) are significantly different (*P* < 0.05, one-way ANOVA). Control, AMN1/10, and AMN1/20 groups were non-challenged groups and exposed to 0, 0.42, and 0.21 mg/L of unionized ammonia, respectively. SH, SH + AMN1/10, and SH + AMN1/20 groups were intraperitoneal challenged with Shewanella spp. and exposed to 0, 0.42, and 0.21 mg/L of ammonium chloride, respectively

ALT and AST levels were significantly elevated (*P* = 0.007) in the SH + AMN1/10, SH + AMN1/20, SH, AMN1/10, and AMN1/20 groups compared to those in the control group (Fig. 5A and B). Compared to those in the control group, the creatinine levels in all groups were significantly different (*P* = 0.033), following the pattern of SH + AMN1/20 > SH + AMN1/10 > SH > AMN1/10 > AMN1/20 (Fig. 5C). Urea levels were significantly greater (*P* = 0.003) in the SH, SH + AMN1/10, and SH + AMN1/20 groups than in the other groups, but there were no significant differences between them (Fig. 5D).

**Fig. 6:**
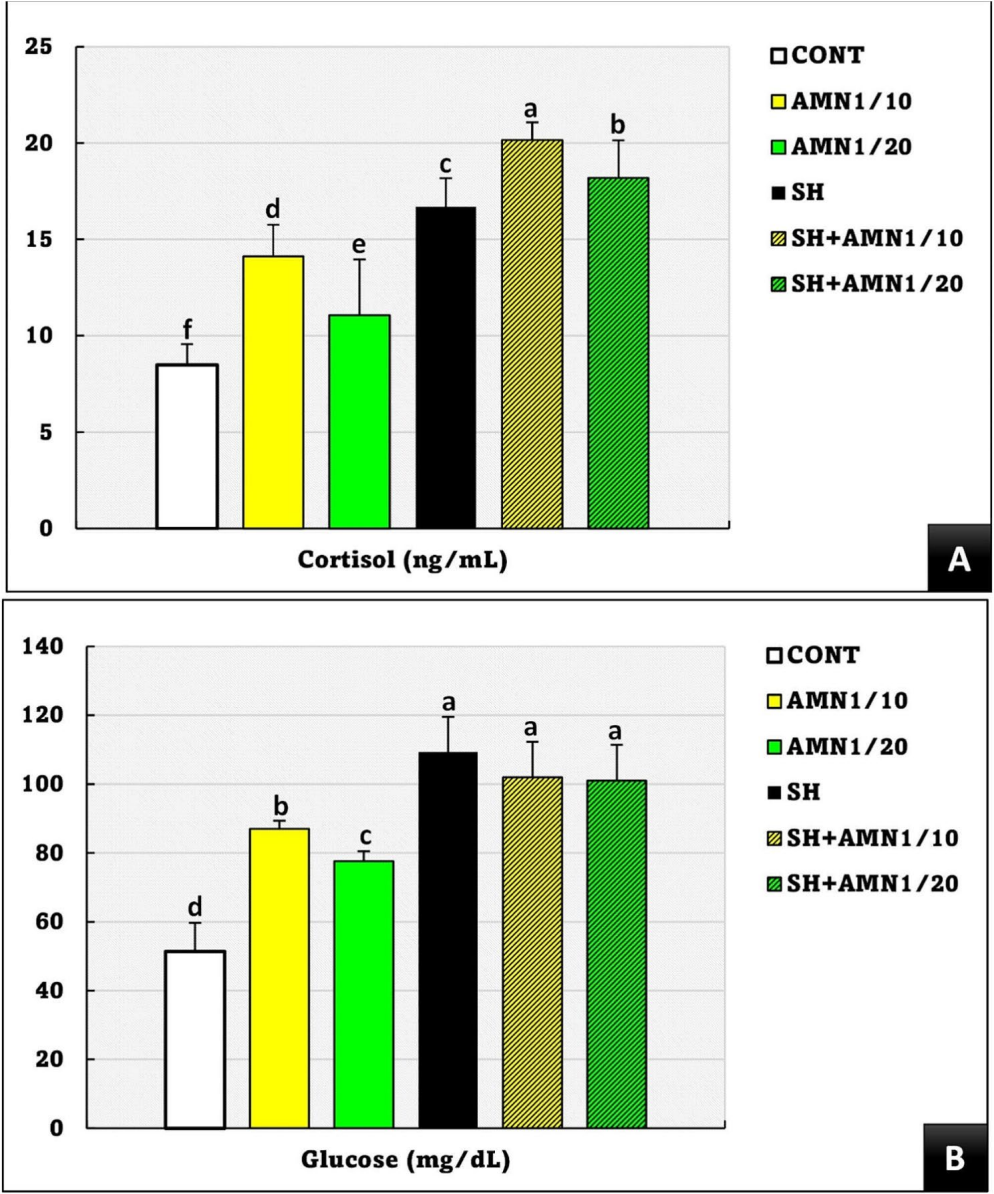
Cortisol (**A**), and glucose (**B**) levels for Nile tilapia challenged with Shewanella spp. and reared under different ammonia levels. Values are presented as the mean ± SE (*n* = 9/group). The bars with different superscripts (**a**, **b**, **c**, **d**, **e**, and **f**) are significantly different (*P* < 0.05, one-way ANOVA). Control, AMN1/10, and AMN1/20 groups were non-challenged groups and exposed to 0, 0.42, and 0.21 mg/L of unionized ammonia, respectively. SH, SH + AMN1/10, and SH + AMN1/20 groups were intraperitoneal challenged with Shewanella spp. and exposed to 0, 0.42, and 0.21 mg/L of ammonium chloride, respectively

Similarly, the cortisol concentrations were significantly different (*P* = 0.04) between the groups, following the pattern of the SH + AMN1/10 > SH + AMN1/20 > SH > AMN1/10 > AMN1/20 compared to those in the control group (Fig. 6A). Glucose levels were significantly elevated (*P* = 0.0001) in the SH + AMN1/10, SH + AMN1/20, and SH groups (without differences between them), followed by the AMN1/10 group, and the AMN1/20 group compared to those in the control group (Fig. 6B).

### Oxidant/antioxidant indices

**Fig. 7:**
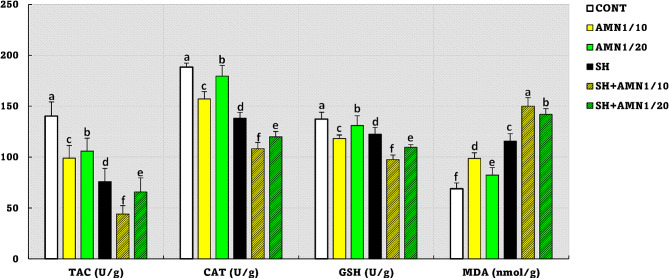
Total antioxidant capacity (TAC), catalase (CAT), reduced glutathione (GSH), and malondialdehyde (MDA) levels for Nile tilapia challenged with Shewanella spp. and reared under different ammonia levels. Values are presented as the mean ± SE (*n* = 9/group). The bars with different superscripts (**a**, **b**, **c**, **d**, **e**, and **f**) are significantly different (*P* < 0.05, one-way ANOVA). Control, AMN1/10, and AMN1/20 groups were non-challenged groups and exposed to 0, 0.42, and 0.21 mg/L of unionized ammonia, respectively. SH, SH + AMN1/10, and SH + AMN1/20 groups were intraperitoneal challenged with Shewanella spp. and exposed to 0, 0.42, and 0.21 mg/L of ammonium chloride, respectively

As shown in Fig. 7, compared with those in the control group, the antioxidant parameters (TAC, CAT, and GSH) were lower (*P* = 0.0001) and the MDA level (*P* = 0.0001) was greater noticed in the following pattern: SH + AMN1/10 < SH < SH + AMN1/20 < AMN1/10 < AMN1/20.

### Immune indices

**Fig. 8:**
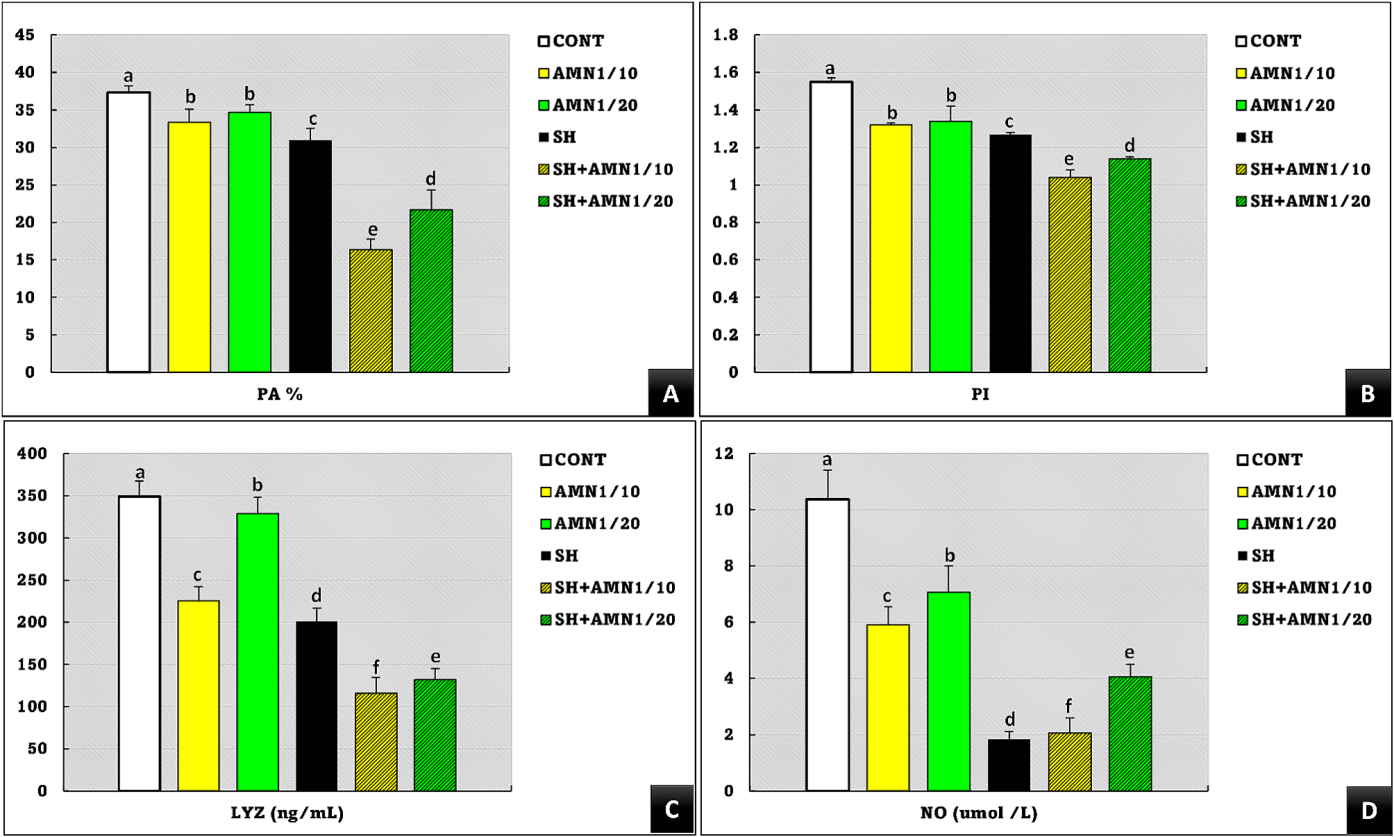
Phagocytic activity (PA%, **A**), phagocytic index (PI, **B**), lysozyme activity (LYZ, **C**), and nitric oxide (NO, **D**) levels for Nile tilapia challenged with Shewanella spp. and reared under different ammonia levels. Values are presented as the mean ± SE (*n* = 9/group). The bars with different superscripts (**a**, **b**, **c**, **d**, **e**, and **f**) are significantly different (*P* < 0.05, one-way ANOVA). Control, AMN1/10, and AMN1/20 groups were non-challenged groups and exposed to 0, 0.42, and 0.21 mg/L of unionized ammonia, respectively. SH, SH + AMN1/10, and SH + AMN1/20 groups were intraperitoneal challenged with Shewanella spp. and exposed to 0, 0.42, and 0.21 mg/L of ammonium chloride, respectively

Figure 8 shows that compared with those in the control group, the PA%, PI, and LYZ activities in the treatment groups were significantly lower (*P* = 0.000, *P* = 0.04, and *P* = 0.01, respectively) in the order SH + AMN1/10 < SH + AMN1/20 < SH. AMN1/10 and AMN1/20 were also significantly lower in PA% and PI than in the control group, whereas the LYZ activity in AMN1/10 was significantly lower than that in AMN1/20. The NO levels were significantly lower (*P* = 0.001) in the SH, SH + AMN1/10, SH + AMN1/20, AMN/10, and AMN1/20groups compared to those in the control group.

**Fig. 9:**
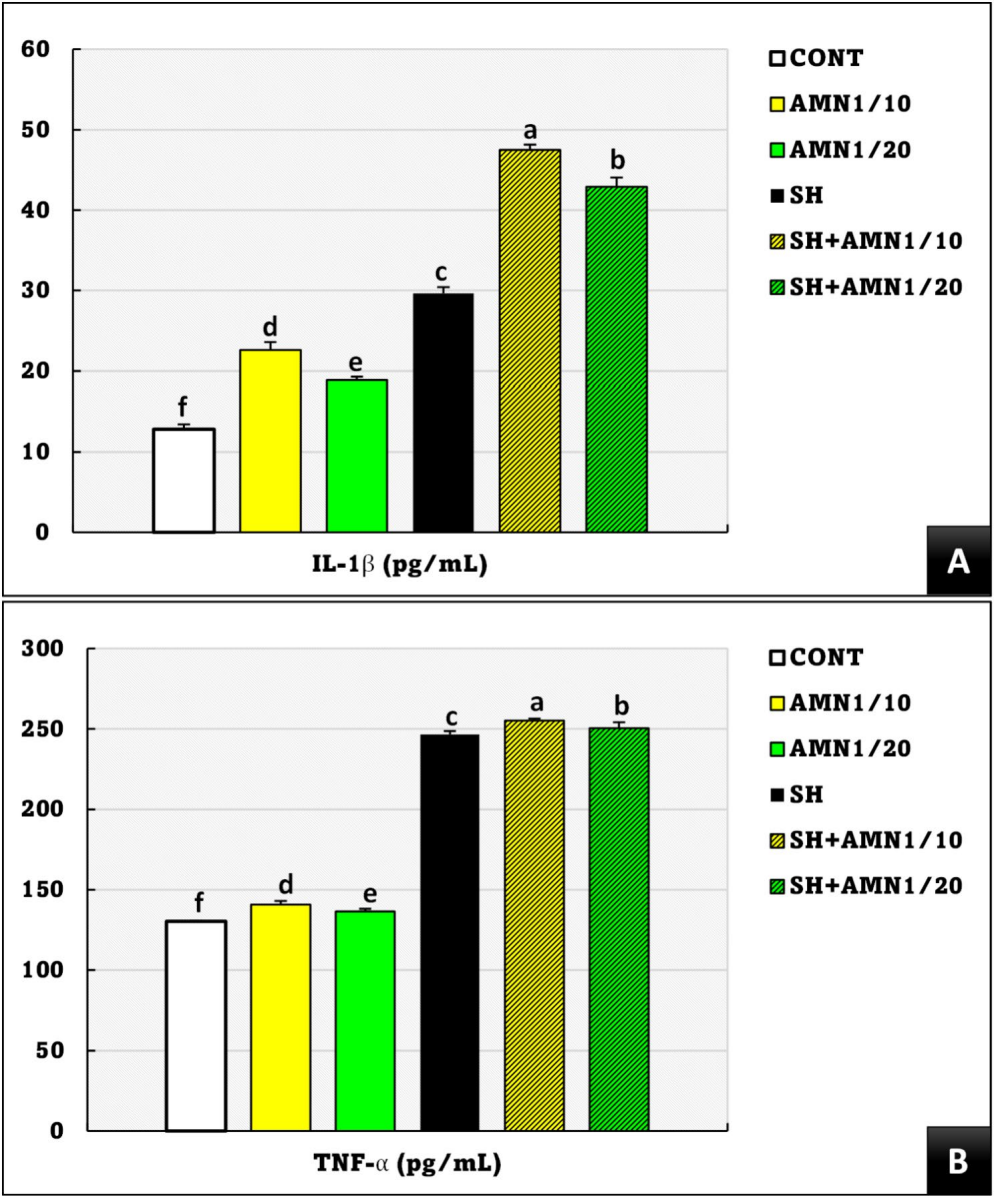
Interleukin-1β (IL-1β, A), and tumor necrosis factor-α (TNF-α, B) for Nile tilapia challenged with Shewanella spp. and reared under different ammonia levels. Values are presented as the mean ± SE (*n* = 9/group). The bars with different superscripts (**a**, **b**, **c**, **d**, **e**, and **f**) are significantly different (*P* < 0.05, one-way ANOVA). Control, AMN1/10, and AMN1/20 groups were non-challenged groups and exposed to 0, 0.42, and 0.21 mg/L of unionized ammonia, respectively. SH, SH + AMN1/10, and SH + AMN1/20 groups were intraperitoneal challenged with Shewanella spp. and exposed to 0, 0.42, and 0.21 mg/L of ammonium chloride, respectively

The IL-β and TNF-α levels were significantly elevated (*P* = 0.0001) in the SH + AMN1/10, SH + AMN1/20, SH, AMN/10, and AMN1/20 groups compared to those in the control group (Fig. 9).

### Histological findings

**Fig. 10:**
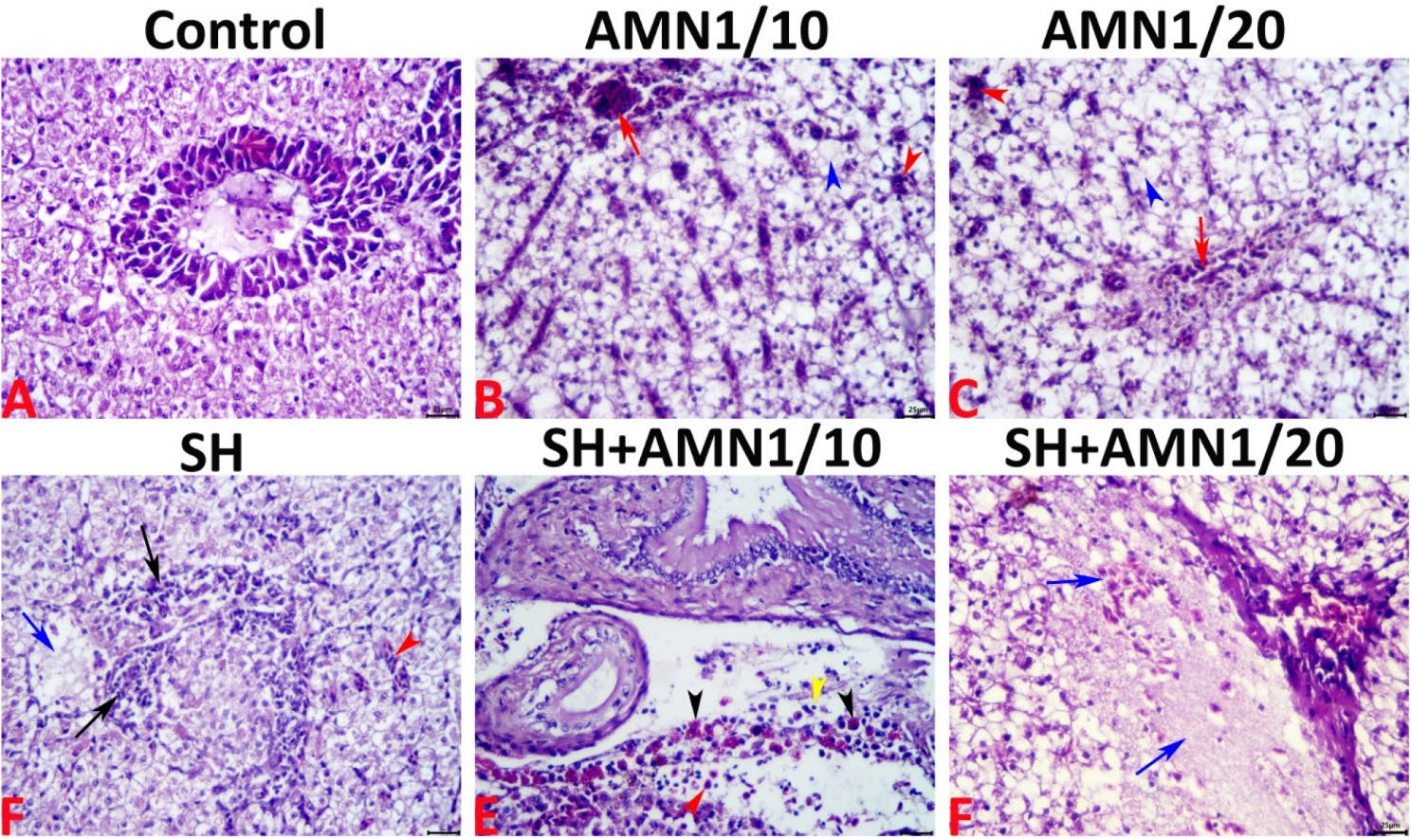
Representative light micrographs of the H&E-stained hepatic tissue sections showing: a normal histological picture in the control group (**A**), acute cellular swelling with notable vacuolations (blue arrowheads), sinusoidal congestions (red arrowheads), and lytic necrotic foci infiltrated by extravasated erythrocytes (red arrows) in the AMN1/10 (**B**), and the AMN1/20 (**C**) groups, sinusoidal (red arrowhead), and vascular (blue arrow). Lytic necrotic foci infiltrated with inflammatory cells (black arrow) in the SH group (**D**), and vascular congestion (red arrowhead), and infiltration with lymphocytes (black arrowhead), and eosinophilic granular cells (black arrowheads) in the SH + AMN1/10 group (**E**) and lytic necrotic foci infiltrated with erythrocytes (blue arrows) in the SH + AMN1/20 group (**F**). The scale bar equals 25 μm

The microscopic examination revealed normal histoarchitectures of the hepatopancreas with no histological alterations in the control group (Fig. 10A). Conversely, exposure to high ammonia concentrations incited an array of morphological alterations, including notable cellular swelling of a lipoidal nature (clear cytoplasm with pushing of the nuclei to the periphery of the cells), congestions of the blood vessels, and sinusoidal capillaries, single cell necrosis, and lytic necrotic foci usually infiltrated by extravasated erythrocytes. These alterations were more severe in the AMN1/10 group (Fig. 10B) (liver index = 15.90 ± 1.64) than in the AMN1/20 group (Fig. 10C) (liver index = 8.90 ± 2.06). In the SH group numerous hepatopathic changes including necrosis of the melanomacrophage centers (MMCs), vascular congestion, inflammatory cell infiltration primarily with mononuclear cells, and lytic necrotic foci usually infiltrated with inflammatory cells (Fig. 10D), (liver index = 16 ± 0.55). Co-exposure to *Shewanella* spp. infection and high levels of ammonia caused significant hepatic damage, as indicated by notable single-cell necrosis, multifocal lytic necrotic foci, large areas of lytic necrosis, and obvious inflammatory cell infiltrates including eosinophilic granule cells. These alterations were more severe in the AMN1/10 + SH group (Fig. 10E), (liver index = 23.70 ± 1.13) than in the AMN1/20 + SH group (Fig. 10F), (liver index = 20.30 ± 1.59). There were no neoplastic, or preneoplastic alterations in the hepatocytes. Additionally, the scattered pancreatic tissue and biliary system showed no significant histological alterations in any of the groups. The details of the hepatopathic alterations and liver indices of all the groups are summarized in Table 3.

**Table 3:** The hepatic and renal histology of *O. niloticus* infected with Shewanella spp and exposed to ammonia

Organ	Histopathological criteria			Control		AMN1/10		AMN1/20		SH		SH+AMN1/10		SH+AMN1/10		*P*- value
	Reaction pattern	Alteration	w	F	Index	F	Index	F	Index	F	Index	F	Index	F	Index	
	Regressive pattern	- Acute cellular swelling	1	0	0.0±0.0	33	0.0±0.0	21	0.0±0.0	11	0.0±0.0	39	0.0±0.0	32	0.0±0.0	-
		- Single-cell necrosis	3	0	0.0±0.0^d^	19	3.0±0.51^b^	11	2.0±0.29^c^	17	0.8±0.20	27	4.0±0.29^a^	21	2.7±0.15^bc^	0
		- Vacuolation foci	2	0	0.0±0.0^c^	7	3.6±0.40^ab^	4	3.0±0.00^b^	3	3.3±0.30^ab^	9	4.2±0.48^a^	5	3.9±0.45^ab^	0
		- Lytic necrotic foci	3	0	0.0±0.0^c^	13	1.4±0.30^ab^	7	0.8±0.32^bc^	15	0.6±0.30^bc^	21	1.8±0.20^a^	19	1±0.30^ab^	0
		- MMCs necrosis	3	0	0.0±0.0^c^	6	3±0.00^ab^	4	2.1±0.45^b^	6	3.3±0.30^a^	14	3.9±0.45^a^	8	3.6±0.40^a^	0
	Inflammatory pattern	- Leukocytic infiltration	2	0	0.0±0.0^d^	7	1.8±0.48^bc^	5	1.2±0.48^c^	20	1.8±0.48^bc^	27	3.0±0.00^a^	25	2.4±0.40^ab^	0
		- Eosinophilic granular cell infiltrate	2	0	0.0±0.0^d^	0	1.4±0.30^c^	0	1±0.33^cd^	3	2.6±0.42^b^	4	3.8±0.55^a^	3	3±0.44^ab^	0
	Circulatory pattern	-Vascular congestion	1	0	0.0±0.0^c^	21	0.0±0.0^c^	15	0.0±0.0^c^	19	0.6±0.30^ab^	31	0.8±0.32^a^	29	0.6±0.30^ab^	0.02
Liver		- Sinusoidal congestion	1	0	0.0±0.0^c^	21	2.60±0.58^ab^	15	1.40±0.22^b^	17	2.30±0.47^ab^	29	3.10±0.52^a^	25	2.70±0.55^ab^	0
		- Hemorrhages	2	0	0.1±0.1^c^	7	2.30±0.15^ab^	6	2.00±0.25^b^	4	1.30±0.15^b^	13	3.10±0.52^a^	11	3.10±0.60^a^	0
	Progressive alterations	- Regenerated hepatocytes	2	0	0.20±0.20^d^	3	1.50±0.34^bc^	3	1.00±0.33^c^	4	0.80±0.32^cd^	5	2.50±0.16^a^	5	2.10±0.10^ab^	0
		- Hyperplastic cholangiocytes	2	0	0.0±0.0^b^	0	0.60±0.30^ab^	0	0.60±0.30^ab^	0	0.80±0.32^ab^	0	1.00±0.33^a^	0	0.80±0.32^ab^	0.02
		- Basophilic foci	1	0	0.0±0.0	0	0.0±0.0	0	0.0±0.0	0	0.0±0.0	1	0.0±0.0	0	0.0±0.0	-
		- MMCs hyperplasia	2	0	0.0±0.0	0	0.20±0.20	0	0.20±0.20	0	0.20±0.20	2	0.20±0.20	2	0.40±0.26	0.83
	Neoplastic alterations	- Epithelial neoplasms	3	0	0.0±0.0	0	0.0±0.0	0	0.0±0.0	0	0.0±0.0	0	0.40±0.26	0	0.40±0.26	0.12
		- Non-epithelial neoplasms	3	0	0.0±0.0	0	0.0±0.0	0	0.0±0.0	0	0.0±0.0	0	0.0±0.0	0	0.0±0.0	-
	Liver index			0.0±0.0^d^		15.90±1.64^b^		8.90±2.06^c^		16±0.55^b^		23.70±1.13^a^		20.30±1.59^a^		0
	Regressive alterations	- Glomerular collapse	2	0	0.0±0.0^c^	13	2.20±0.20^a^	7	1.40±0.30^b^	9	1.80±0.20^ab^	15	2.40±0.26^a^	14	2.00±0.00^ab^	0
		- Glomerular necrosis	3	0	0.0±0.0^d^	13	3.00±0.00^ab^	5	1.50±0.50^c^	6	1.80±0.48^c^	11	3.30±0.30^a^	7	2.10±0.45^bc^	0
		- Tubular vacuolation	1	0	0.0±0.0	39	2.30±0.36^b^	26	1.00±0.00^c^	21	1.00±0.00^c^	41	3.10±0.52^a^	32	1.40±0.22^c^	0
		- Tubular necrosis	3	0	0.0±0.0^c^	19	3.90±0.45^a^	8	2.40±0.40^b^	11	3.00±0.00^ab^	21	3.60±0.40^a^	17	3.30±0.30^ab^	
		- Tubular dilatation	2	0	0.0±0.0^c^	5	1.00±0.33^ab^	4	0.80±0.32^abc^	2	0.40±0.26^bc^	7	1.40±0.30^a^	6	1.20±0.32^ab^	0.01
		- Cast formation	2	0	0.0±0.0^d^	7	1.40±0.30^bc^	3	0.80±0.32^c^	9	1.80±0.20^ab^	15	2.40±0.26^a^	11	2.00±0.00^ab^	0
		- MMCs necrosis	3	0	0.0±0.0^c^	6	1.80±0.48^a^	2	0.60±0.40^bc^	5	1.50±0.50^ab^	9	2.70±0.30^a^	7	2.10±0.45^a^	0
		- Tunica media vacuolations	2	0	0.0±0.0^b^	4	0.80±0.32^ab^	1	0.20±0.20^ab^	3	0.60±0.30^ab^	5	1.00±0.33^a^	5	1.00±0.33^a^	
	Inflammatory alterations	- Leukocytic infiltration	2	0	0.0±0.0^b^	6	1.20±0.32^b^	2	0.40±0.26^b^	22	3.20±0.44^a^	31	4.20±0.55^a^	23	3.80±0.55^a^	0.05
kidney		- Eosinophilic granular cell infiltrate	2	0	0.0±0.0	0	0.0±0.0	0	0.0±0.0	0	0.0±0.0	0	0.0±0.0	0	0.0±0.0	0
	Circulatory alterations	- Vascular congestion	1	0	0.0±0.0^d^	17	1.50±0.16^bc^	11	1.00±0.00^c^	21	2.30±0.15^a^	32	2.40±0.47^a^	24	2.00±0.33^ab^	-
		- Interstitial hemorrhage	2	0	0.0±0.0^b^	6	1.20±0.32^a^	5	1.20±0.32^a^	4	0.80±0.32^ab^	7	1.40±0.30^a^	7	1.40±0.30^a^	0
		- Interstitial edema	2	0	0.0±0.0^c^	4	0.80±0.32^b^	2	0.60±0.30^bc^	7	1.60±0.26^a^	9	1.80±0.20^a^	8	1.60±0.26^a^	0.009
	Progressive alterations	- Regenerated tubular epithelium	2	0	0.0±0.0^c^	7	1.40±0.30^a^	2	0.40±0.26^bc^	3	0.60±0.30^abc^	5	1.00±0.33^ab^	4	0.80±0.32^abc^	0
		- Endothelial hypertrophy	1	0	0.0±0.0^c^	9	0.90±0.10^a^	3	0.30±0.15^b^	2	0.20±0.13^b^	14	1.00±0.00^a^	7	0.70±0.15^a^	0.19
		- Endothelial hyperplasia	2	0	0.0±0.0	0	0.0±0.0	0	0.20±0.20	0	0.0±0.0	2	0.40±0.26	2	0.20±0.20	0
		- MMCs hyperplasia	2	0	0.0±0.0	0	0.0±0.0	0	0.0±0.0	0	0.0±0.0	2	0.0±0.0	1	0.0±0.0	0.39
	Neoplastic alterations	- Epithelial neoplasms	3	0	0.0±0.0	0	0.0±0.0	0	0.0±0.0	0	0.0±0.0	0	0.0±0.0	0	0.0±0.0	0.2
		- Non-epithelial neoplasms	3	0	0.0±0.0	0	0.0±0.0	0	0.0±0.0	0	0.0±0.0	0	0.0±0.0	0	0.0±0.0	-
	Kidney index			0.0±0.0^e^		21.40±0.96^c^		15.30±2.01^d^		18.40±1.52^cd^		31.80±1.29^a^		26.70±1.22^b^		0

F, alteration frequency; W, importance factor; LI, liver Index; KI, Kidney Index. The values are shown in means ± SE. The means within the same row carrying different superscripts are significant at *P*<0.05

**Fig. 11:**
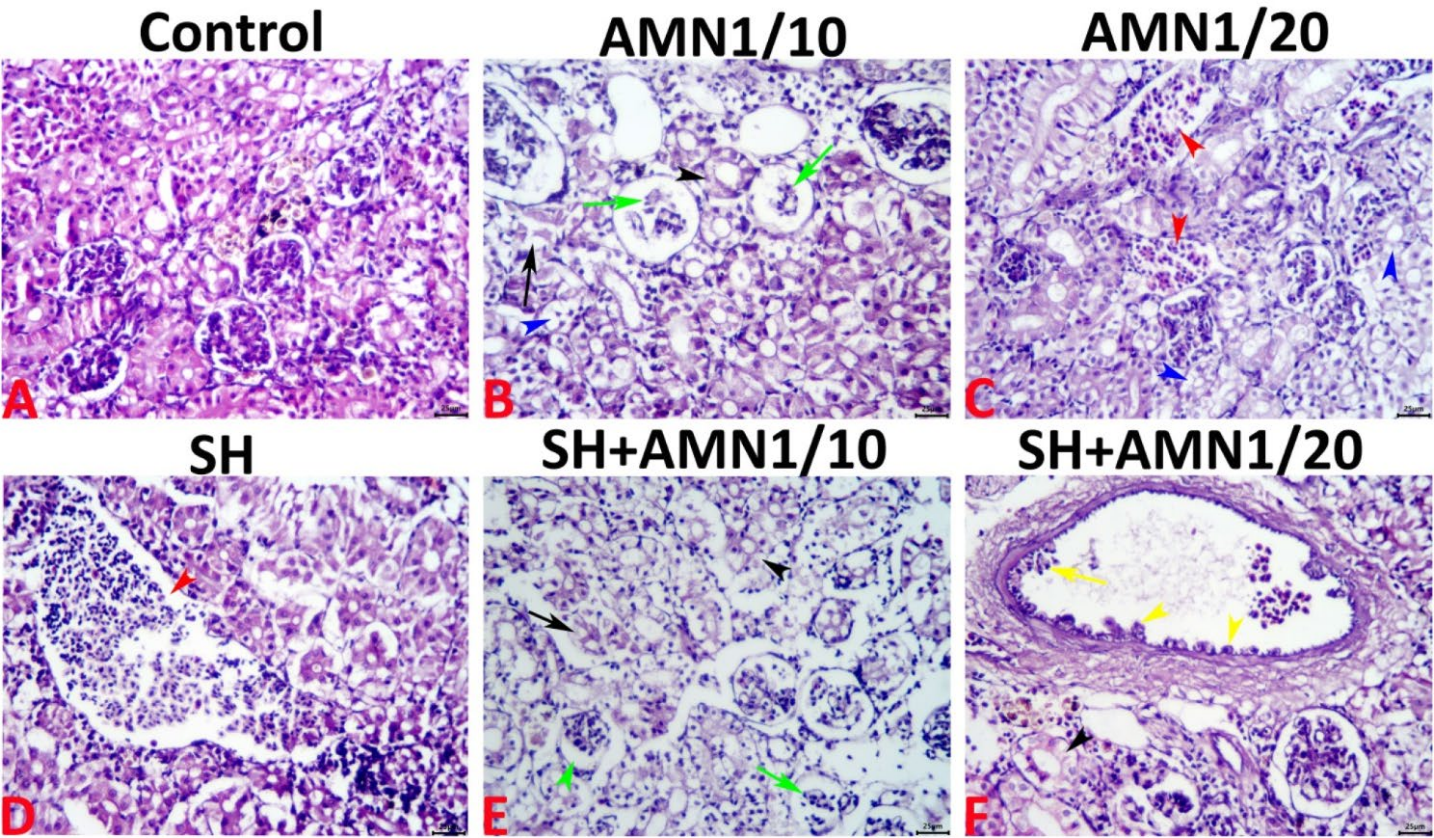
Representative light micrographs of the H&E-stained renal tissue sections showing normal histological picture in the control group (**A**), glomerular necrosis (green arrows), single-cell necrosis (blue arrowhead), and complete tubular necrosis (black arrow) in the AMN1/10 group (**B**), necrotic MMCs (black ellipses), vacuolated tubular epithelium (blue arrowheads), and vascular congestions (red arrowheads) in the AMN1/20 group (**C**). Vascular congestion (red arrowhead), and inflammatory cell infiltrate (red ellipse) in the SH group (**D**). Glomerular necrosis (green arrow), and collapse with widen Bowman’s space (green arrowhead), tubular single-cell necrosis (black arrowhead), and complete tubular necrosis (black arrow) in the SH + AMN1/10 group (**E**). Glomerular necrosis (green arrowhead), tubular single-cell necrosis (black arrowhead), and vascular congestion associated with endothelial hypertrophy (yellow arrowhead), and hyperplasia (yellow arrow), hyalinization (red arrowhead), and vacuolations (blue arrowheads) of the tunica media in the SH + AMN1/20 group (**F**). The scale bar equals 20 μm

Normal histology with no histological alterations was evident in the renal tissue specimens of the control group (Fig. 11A). Kidneys from the AMN1/10 group exhibited a vast array of morphological changes, most importantly, notable vacuolation and necrosis of the tubular epithelium, and glomerular necrosis (Fig. 11B), (kidney index = 21.40 ± 0.96). However, the kidneys of the AMN1/20 group exhibited some nephropathic alterations such as vascular congestion, vacuolation of the tubular epithelium, and necrosis of the MMCs (Fig. 11C), (kidney index = 15.30 ± 2.01). Kidneys from the SH group exhibited a few nephropathic alterations represented by vascular congestion, and inflammatory cell infiltrates of a mononuclear nature (Fig. 11D), (kidney index = 18.40 ± 1.52). Infection with *Shewanella* spp. in combination with high levels of ammonia in water exacerbated kidney cell injury in the AMN1/10 + SH and AMN1/20 + SH groups. In both groups, tubular vacuolations, necrosis, glomerular lobulations, necrosis, interstitial inflammatory cell infiltration, vascular congestion, endothelial hypertrophy, and hyperplasia are accompanied by hyalinization and vacuolations of the tunica media. Kidney cell injury was more severe in the AMN1/10 + SH group (Fig. 11E), (kidney index = 31.80 ± 1.29) than in the AMN1/20 + SH group (Fig. 11F), (kidney index = 26.70 ± 1.22). The details of the nephropathic alterations and kidney indices of all the groups are summarized in Table 3.

## Discussion

Understanding and maintaining ammonia levels are crucial in aquaculture systems. Fish excretion, uneaten feed decomposition, and high stocking density in aquaculture systems all contribute to ammonia [52]. Most ammonia exists in a harmless form (ionized ammonia, NH_4_). However, a portion of ammonia exists as unionized ammonia (NH_3_), which is highly toxic to fish [53, 54]. When fish are stressed due to factors such as unionized ammonia, their immune system becomes less effective. This creates an opportunity for opportunistic bacteria present in the water to invade the fish and cause disease. Stress can also alter the internal environment of fish, increasing susceptibility to bacterial growth and potentially even increasing the virulence (harmfulness) of certain bacteria [10, 15, 55–57]. Some studies have linked the outbreak of some diseases in aquatic systems to the quality of water parameters [14, 58–61]. A meta-analysis by Paredes-Trujillo and Mendoza-Carranza [59] revealed a significant association between ammonia (NH_3_) and infectious diseases in 41% of the investigated publications.

This study revealed similar symptoms in the AMN1/10 and AMN1/20 groups, but more severe symptoms in the AMN1/10 group. Symptoms included skin color change to dark coloration, fin destruction; lethargy, open mouths, excessive mucus secretion, swelling, and congestion in the gills. The detrimental effects of ammonia on muscle membranes and metabolism, which can result in lethargy, could explain the recorded symptoms. Additionally, the damaging impact of ammonia on gill filaments can result in excessive mucus secretion, which can cause respiratory distress [11, 12, 53]. In another study, *O. niloticus* newly hatched larvae exposed to 0.5–0.6 mg/l UIA exhibited signs of evidence of losing equilibrium, swimming sideways, and attempting to breathe oxygen. Some were pale and had excess mucus in their skin and gills [62]. Furthermore, hyperactivity and rapid operculation were recorded in O. niloticus exposed to 0.5 mg/L UIA after 120 h of exposure [55]. Variations in fish response and severity of signs of ammonia toxicity across studies can stem from differences in ammonia concentration, exposure duration, fish species and their life stages, and water quality parameters. In the present study, the SH group exhibited severe fin rot, lower jaw hemorrhage, and ascites in some cases. The virulence factors of *Shewanella* species, including their capacity to release cytotoxins, adhere to host cells, and produce siderophores, may be attributed to the appearance of these symptoms [20]. However, the SH + AMN1/10 group in this study showed more severe symptoms, including scale loss, mucus secretion, ulcers, expanded gill cover, and fish death with an open mouth with congestion in the internal organs. In a similar study, Abdel-Latif, Shukry [56] reported that exposure of *O. niloticus* to both ammonia and *Aeromonas hydrophila* simultaneously worsened the severity of this disease. Research on the impact of bacterial infection in the presence of ammonia stress on fish survival has been inconsistent. Several studies agree with us that bacterial infection in the presence of ammonia stress has a negative effect on the survival rates of fish [14, 18, 56]. Abreu, Magalhães [63] reported that this might be because certain bacteria, such as those in the Aeromonadaceae family, use ammonia as a growth factor, which promotes their proliferation and virulence. On the contrary, Farmer, Mitchell [64] reported that immersing fish in NH_4_Cl at a concentration of 46.3 mg/L decreased the mortality rate of *Flavobacterium columnare* infected fish. The authors of this study have proposed several hypotheses, the most important of which is that ammonia hampers the ability of bacteria to attach to the fish body.

Hematological examination is crucial in assessing fish health following exposure to environmental stressors like ammonia [65]. Changes in red blood cell counts, hemoglobin content, and white blood cell counts can indicate anemia, infections, or immune system stress. These alterations can be linked to disease or exposure to pollutants in the water, making blood analysis a valuable tool for monitoring fish health and water quality [66, 67]. Therefore, examining these hematological parameters in *Shewanella* spp.-challenged *O. niloticus* exposed to ammonia stress provides valuable insights into the combined effects of these stressors on fish health. Unexpectedly, the hematological indices measured in this study showed no differences among the control, AMN1/10, and AMN1/20 groups. The study by Handayani, Soegianto [68] stated that *O. niloticus* exposed to mercury (Hg) at concentrations of 0.1 and 1 mg/L showed considerably higher levels of RBC, WBC, and Hb; however, only for the 0.1 mg/L dosage did these values recover to control levels after 7 days of exposure. Exposure to mercury did not alter the levels of Ht. This finding aligns with the findings of *Salmo salar* blood Hct, RBC, and MCV, which were unaffected by exposure to low ammonia concentrations (22 µg/L NH_3_-N) for 14–15 days [69].Therefore, this result could be attributed to the low concentrations of ammonia to which the fish were exposed, and the fish may require longer-term stressor exposure. In addition, the fish compensating mechanisms [70] could be the reason for the reported lack of direct effects of NH_3_ on hematological markers. This was corroborated by Witeska, Kondera [65] who mentioned that hematological variables may demonstrate either compensating or destructive consequences of toxicity exposure. Therefore, it is challenging to draw conclusions about the mechanisms underlying the hematological alterations that fish exposed to toxicity frequently exhibit since they are a general, nonspecific stress response. Moreover, in the present study, exposure to UIA at various concentrations and *Shewanella* spp. challenge had the lowest harmful effect on hematological parameters compared with exposure to *Shewanella* spp. challenge alone. This finding could be attributed to the fact that fish were exposed to *Shewanella* challenge simultaneously as they were exposed to ammonia, so the ammonia concentration did not negatively influence the hematological parameters. Farmer, Mitchell [64] made a similar attribution about the combined impact of ammonia exposure and the *F. columnare* challenge on channel catfish survival rate. Prior research has shown that infection by *Shewanella* spp. has harmful impacts on the blood parameters of fish [33]. This could be attributed to the secretion of protease and hemolysin enzymes by *Shewanella* spp., which can cause damage to hemopoietic organs [71, 72].

The liver and kidneys are vital organs in fish that play a crucial role in detoxification and waste removal. As a result, their function can serve as a valuable indicator of fish health. Changes in liver and kidney function can signal exposure to toxins or pollutants in the water, making them ideal biomarkers for aquatic toxicology [73, 74]. Additionally, altered liver and kidney function can be indicative of infectious diseases in fish [33]. In this study, there was a positive correlation between UIA concentration and the levels of ALT and AST in *Shewanella*-challenged groups, which presented the highest ALT and AST levels compared to those in the control group. Furthermore, compared to the control group, the SH group and *Shewanella*-challenged groups exposed to UIA had greater creatinine and urea levels than the groups exposed to UIA alone without challenge. These results indicate that co-exposure to ammonia stress and *Shewanella* infection can have a synergistic effect on the liver and kidney functions of fish. Previous studies have proven that ammonia can damage the liver and kidneys, which are essential organs for detoxification and waste removal [10, 12, 53]. Additionally, other studies have shown alterations in liver and kidney function in *Shewanella*-infected fish, and some have attributed these changes to virulence factors and toxins [22, 25, 33]. Therefore, the combined effects of both ammonia stress and *Shewanella* infection on *O. niloticus* may be more severe than the effects of either stressor alone.

Cortisol and glucose levels serve as common indicators of stress in fish [75, 76]. An increase in cortisol and ammonia levels has been documented in previous research in response to individual exposure to ammonia stress [12, 77, 78] or *Shewanella* infection [33]. By examining cortisol and glucose levels; we can better understand the interplay between stress response, *Shewanella* spp. infection, and metabolic function in *Oreochromis niloticus* exposed to these stressors. The present results corroborate that co-exposure to ammonia stress and *Shewanella* infection (SH + AMN1/10 and SH + AMN1/20) had synergistic effects on the levels of cortisol and glucose, which were greater than those in the control group. Evans, Pasnik [55] reported that *O. niloticus* in both the UIA-exposed and control groups exhibited physiologic responses to *Streptococcus agalactiae* challenge via increased glucose levels. The researchers attributed this result to either handling stress or infection. On the other hand, Benli and Yildiza [79] suggested that the glucose stress response in Nile tilapia infected with *Edwardsiella tarda* was due to poor water quality conditions, not infection.

Fish exposed to both *Shewanella* infection and ammonia stress face dual stress. Bacterial infections ramp up the production of harmful molecules called reactive oxygen species (ROS) within fish cells [80, 81]. Ammonia exposure also disrupts cellular balance and causes ROS production to overdrive [82–84]. Furthermore, the increase in stress factors (cortisol and glucose) in this study plays a major role in the production of ROS by activating an enzyme called NADPH oxidase that generates ROS directly [85, 86]. To combat this oxidative stress, fish activate antioxidant defenses, including enzymes such as TAC, CAT, and GSH. TAC is one of the most crucial metrics because it provides a holistic evaluation of the total antioxidant status of fish samples by providing a thorough assessment of the cumulative effect of all antioxidants, both enzymatic and non-enzymatic [87]. Catalase plays a critical role in the breakdown of hydrogen peroxide, a common reactive oxygen species (ROS), into water and oxygen [88]. Glutathione, a non-enzymatic antioxidant, is essential for cellular redox equilibrium because it scavenges free radicals and serves as a substrate for antioxidant enzymes [89]. These antioxidants work to neutralize ROS and protect cells from damage. However, the combined factors, ammonia stress and *Shewanella* infection, can disrupt the antioxidant system, as demonstrated in the present study. The antioxidant indices (TAC, CAT, and GSH) decreased significantly with increasing oxidant concentrations (MDA) in either group only exposed to the UIA or simultaneously infected and exposed to UIA (SH + AMN1/10 and SH + AMN1/20). These findings demonstrate that fish exposed to dual stress (ammonia and *Shewanella* infection) experience oxidative stress. This finding supports the explanations for the altered liver and kidney functions in these study groups. In contrast, the expression of SOD genes in Nile tilapia increased under ammonia stress and *A. hydrophila* infection, while glutathione-S-transferase (GST) gene expression decreased in hepatic tissues exposed to single or dual stressors [56]. Several variables can impact the results, including variations in experimental technique, type of fish and bacteria used, level of ammonia, duration of exposure, organs analyzed, and timing of sample collection among different investigations [81].

Phagocytosis is the mechanism by which a cell engulfs various particle targets. Lysozyme is crucial for breaking down bacterial cell walls [90]. On the other hand, NO is a signaling molecule with antimicrobial properties. It’s produced by immune cells to combat pathogens [91]. IL-1β and TNF-α are pro-inflammatory cytokines that orchestrate the immune response [92]. By examining these parameters, we can establish a more comprehensive understanding of how ammonia stress influences the fish’s immune response to *Shewanella* spp. infection. In this study, the fish groups exposed to the combined stress of ammonia and *Shewanella* infection (SH + AMN1/10, then SH + AMN1/20) had the lowest levels of immune parameters (PI, PA%, and LYZ) and the highest levels of proinflammatory cytokines (IL-1β and TNF-α). Cortisol, an important regulator of the neuro-immunoendocrine system, is associated with immune suppression in fish because it affects the hypothalamic-pituitary-interrenal axis [93]. Our results can be explained by the evidence that shows a correlation between elevated levels of cortisol in the bloodstream and a decrease in immune parameters (PI, PA%, and LYZ) [94]. The degree of suppression can vary depending on the severity and duration of the stress and the specific fish species. Several studies suggest that chronic stress can lead to long-term impairments in fish immune function [95–97]. Abdel-Latif, Shukry [56] reported heightened levels of proinflammatory cytokines and CXC chemokines in the kidney tissues of *O. niloticus* subjected to ammonia stress or *A. hydrophila*. These findings suggest that the immune system of fish responds by increasing the levels of these substances to reduce inflammatory reactions.

The liver and kidneys are crucial organs for detoxification but are vulnerable to water pollutants such as ammonia, suggesting that they are targets for ammonia poisoning [10]. Numerous papers document histopathological changes in the liver and kidney of ammonia-exposed fish, the extent of which these changes vary based on the concentration and exposure period [10, 56, 98, 99]. Additionally, different kinds of fish exhibit histological changes in their internal organs, mostly in the liver and kidneys [24, 25, 33]. These alterations are mainly due to the enzyme activity of *Shewanella* infection, ability to adhere to cells, and the release of cytotoxins [20]. In the present study, high ammonia exposure caused morphological alterations, including lipoidal swelling, blood vessel congestion, single cell necrosis, and lytic necrotic foci. These alterations were more severe in the AMN1/10 and SH groups. Co-exposure to *Shewanella* spp. infection and high ammonia concentrations cause significant hepatic damage, with single-cell necrosis, multifocal lytic necrotic foci, and inflammatory cell infiltrates. Additionally, the kidneys of the AMN1/10 group exhibited many changes in shape, such as tubular epithelium vacuolations and necrosis, glomerular necrosis, and vascular congestion. The kidneys of AMN1/20 also exhibited nephropathic alterations. Infection with *Shewanella* spp. and high ammonia levels in the AMN1/10 + SH and AMN1/20 + SH groups worsened kidney cell damage. Similarly, *O. niloticus* exposed to dual stress (ammonia and *A. hydrophila* infection) displayed vacuolation of hepatocytes, protuberant fatty degeneration, and periacinar necrosis in hepatopancreatic tissues. However, the posterior kidney of these fish displayed infiltration of inflammatory cells with severe necrosis and degeneration [56].

## Conclusion

This study indicated that ammonia exposure synergistically affects *Shewanella* infection in Nile tilapia. In the groups co-exposed to ammonia and *Shewanella* infection, there was a significant increase in the mortality rate and stress markers (cortisol and glucose levels) and changes in liver and kidney function and structure. Conversely, significant decreases in hematological indices, antioxidant activity, and immunological parameters were observed. Accordingly, these results thoroughly grasp the *Shewanella* infection in Nile tilapia and the role that ammonia stress plays in those infections. The results also highlighted the need for water quality maintenance to protect the aquaculture sector against these opportunistic bacteria.

## Data Availability

All data generated or analyzed during this study are included in this published article.

## References

[1] Troell M, Costa-Pierce B, Stead S, Cottrell RS, Brugere C, Farmery AK (2023). Perspectives on aquaculture’s contribution to the Sustainable Development Goals for improved human and planetary health. J World Aquac Soc.

[2] Yarkina NN, Logunova NN. Fisheries and aquaculture: implementing Sustainable Development Goals. Sustainable fisheries and aquaculture: challenges and prospects for the Blue Bioeconomy. Springer; 2022. pp. 149–60.

[3] Atukunda P, Eide WB, Kardel KR, Iversen PO, Westerberg AC. Unlocking the potential for achievement of the UN Sustainable Development Goal 2 - ‘Zero Hunger’ - in Africa: targets, strategies, synergies and challenges. Food Nutr Res. 2021;65. 10.29219/fnr.v65.7686 PMC8254460 34262413

[4] Muzari W (2016). Small scale fisheries and fish farming, processing and marketing in sub-saharan Africa: implications for poverty alleviation, food security and nutrition. Int J Sci Res.

[5] Froehlich HE, Runge CA, Gentry RR, Gaines SD, Halpern BS. Comparative terrestrial feed and land use of an aquaculture-dominant world. Proceedings of the National Academy of Sciences. 2018;115(20):5295 – 300. 10.1073/pnas.1801692115 PMC5960322 29712823

[6] MacLeod M, Hasan MR, Robb DHF, Mamun-Ur-Rashid M. Quantifying and mitigating greenhouse gas emissions from global aquaculture. Food and Agriculture Organization of the United Nations; 2019.

[7] Liu F-G, Yang S-D, Chen H-C (2009). Effect of temperature, stocking density and fish size on the ammonia excretion in palmetto bass (*Morone saxatilis*×*M. chrysops*). Aquac Res.

[8] Munguti JM, Kirimi JG, Obiero KO, Ogello EO, Kyule DN, Liti DM (2020). Aqua-feed wastes: impact on natural systems and practical mitigations—A review. J Agric Sci.

[9] Al-Zahrani MS, Hassanien HA, Alsaade FW, Wahsheh HAM. Sustainability of growth performance, Water Quality, and Productivity of Nile Tilapia-Spinach affected by feeding and fasting regimes in Nutrient Film technique-based aquaponics. Sustain [Internet]. 2024; 16(2).

[10] Xu Z, Cao J, Qin X, Qiu W, Mei J, Xie J. Toxic effects on Bioaccumulation, Hematological Parameters, oxidative stress, Immune responses and tissue structure in Fish exposed to Ammonia Nitrogen: a review. Anim [Internet]. 2021; 11(11). 10.3390/ani11113304 PMC8614401 34828036

[11] Randall DJ, Tsui TKN (2002). Ammonia toxicity in fish. Mar Pollut Bull.

[12] Parvathy AJ, Das BC, Jifiriya MJ, Varghese T, Pillai D, Rejish Kumar VJ (2023). Ammonia induced toxico-physiological responses in fish and management interventions. Reviews Aquaculture.

[13] Lin W, Luo H, Wu J, Hung T-C, Cao B, Liu X et al. A review of the emerging risks of Acute Ammonia Nitrogen toxicity to aquatic decapod crustaceans. Water [Internet]. 2023; 15(1).

[14] Abu-Elala NM, Abd-Elsalam RM, Marouf S, Abdelaziz M, Moustafa M, Eutrophication (2016). Ammonia Intoxication, and infectious diseases: interdisciplinary factors of Mass mortalities in cultured Nile Tilapia. J Aquat Anim Health.

[15] Li H, Li Q, Wang S, He J, Li C (2023). Ammonia nitrogen stress increases susceptibility to bacterial infection via blocking IL-1R–Relish axis mediated antimicrobial peptides expression in shrimp. Aquaculture.

[16] Carballo M, Munoz MJ, Cuellar M, Tarazona JV (1995). Effects of waterborne copper, cyanide, ammonia, and nitrite on stress parameters and changes in susceptibility to saprolegniosis in rainbow trout (*Oncorhynchus mykiss*). Appl Environ Microbiol.

[17] Lu X, Luan S, Dai P, Luo K, Chen B, Cao B (2019). Insights into the molecular basis of immunosuppression and increasing pathogen infection severity of ammonia toxicity by transcriptome analysis in pacific white shrimp Litopenaeus vannamei. Fish Shellfish Immunol.

[18] dos Santos Gonçalves AF. Effects of ammonia on the disease susceptibility of fish. 2013.

[19] Satomi M, Rosenberg E, DeLong EF, Lory S, Stackebrandt E, Thompson F (2014). The Family Shewanellaceae. The prokaryotes: Gammaproteobacteria.

[20] Paździor E (2016). Shewanella putrefaciens–a new opportunistic pathogen of freshwater fish. J Veterinary Res.

[21] Tseng SY, Liu PY, Lee YH, Wu ZY, Huang CC, Cheng CC (2018). The pathogenicity of *Shewanella algae* and ability to tolerate a wide range of temperatures and Salinities. Can J Infect Dis Med Microbiol.

[22] El-Barbary MI (2017). First recording of *Shewanella putrefaciens* in cultured *Oreochromis niloticus* and its identification by 16Sr RNA in Egypt. Egypt J Aquat Res.

[23] Kozinska A, Pekala A (2004). First isolation of Shewanella putrefaciens from freshwater fish-a potential new pathogen of fish. Bulletin-European Association Fish Pathologists.

[24] Sood N, Pradhan PK, Ravindra, Verma DK, Yadav MK, Mishra RK (2020). Large-scale mortality in cultured tilapia *Oreochromis Niloticus* due to infection with *Shewanella putrefaciens* in India. J World Aquac Soc.

[25] Jiang X, Wang X, Li L, Niu C, Pei C, Zhu L (2022). Identification of *Shewanella putrefaciens* as a novel pathogen of the largemouth bass (*Micropterus salmoides*) and histopathological analysis of diseased fish. Front Cell Infect Microbiol.

[26] Korun J, Akgun-Dar K, Yazici M (2009). Isolation of *Shewanella putrefaciens* from cultured European sea bass,(*Dicentrarchus labrax*) in Turkey. Rev Med Vet.

[27] Nickum JG, Bart HL, Bowser PR, Greer IE, Jenkins JA, MacMillan JR (2015). Guidelines for the Use of fishes in Research (2004).

[28] Johansen R, Needham JR, Colquhoun DJ, Poppe TT, Smith AJ (2006). Guidelines for health and welfare monitoring of fish used in research. Lab Anim.

[29] APHA (1998). Standard methods for the examination of water and wastewater.

[30] NRC. National Research Council. Nutrient requirements of fish and shrimp. National academies; 2011.

[31] Almazán-Rueda P, Schrama JW, Verreth JAJ (2004). Behavioural responses under different feeding methods and light regimes of the African catfish (*Clarias gariepinus*) juveniles. Aquaculture.

[32] Wang Y, Guo J-l, Li K, Bureau DP (2006). Effects of dietary protein and energy levels on growth, feed utilization and body composition of cuneate drum (*Nibea miichthioides*). Aquaculture.

[33] Reda RM, El-Murr A, Abdel-Basset NA, Metwally MMM, Ibrahim RE (2024). Infection dynamics of Shewanella spp. in Nile tilapia under varied water temperatures: a hematological, biochemical, antioxidant-immune analysis, and histopathological alterations. Fish Shellfish Immunol.

[34] Raiesi SN, Mazandarani M, Shahidi S-A, Ghorbani-HasanSaraei A, Khani F, Fatahi S (2018). Acute and chronic toxicity of ammonia in Persian sturgeon, *Acipenser persicus*, fingerlings. Int J Aquat Biology.

[35] Kucuk S. The effect of Diel unionized ammonia fluctuation on juvenile hybrid striped bass, channel catfish, and tilapia. Mississippi State University; 1999.

[36] OECD. Guideline for Testing of Chemicals 203 Fish, Acute Toxicity Test. Organisation for Economic Cooperation and Development. Paris, France; 1992.

[37] Finney DJ (1971). A statistical treatment of the sigmoid response curve.

[38] Neiffer DL, Stamper MA (2009). Fish Sedation, Anesthesia, Analgesia, and Euthanasia: considerations, methods, and types of drugs. ILAR J.

[39] Lu X, Ji Z-H, Wang M-Z, Tian J, Dong L-X, Guo Z-B (2023). The requirement and Protective effects of Dietary protein against chronic Ammonia exposure in Juvenile genetically Improved Farmed Tilapia (*Oreochromis niloticus*). Aquacult Nutr.

[40] Tran-Duy A, Schrama JW, van Dam AA, Verreth JA (2008). Effects of oxygen concentration and body weight on maximum feed intake, growth and hematological parameters of Nile tilapia, Oreochromis niloticus. Aquaculture.

[41] Jain NC. Essentials of veterinary hematology 1993.

[42] Fossati P, Prencipe L, Berti G (1983). Enzymic creatinine assay: a new colorimetric method based on hydrogen peroxide measurement. Clin Chem.

[43] Saliu J, Oluberu S, Akpoke I, Ukwa UJAJAS. Cortisol stress response and histopathological alteration index in Clarias gariepinus exposed to sublethal concentrations of Qua Iboe crude oil and rig wash. 2017;42(1):55–64.

[44] Trinder P (1969). Determination of blood glucose using 4-amino phenazone as oxygen acceptor. J Clin Pathol.

[45] Reda RM, Helmy RMA, Osman A, Ahmed FAG, Kotb GAM, El-Fattah AHA (2023). The potential effect of Moringa oleifera ethanolic leaf extract against oxidative stress, immune response disruption induced by abamectin exposure in Oreochromis niloticus. Environ Sci Pollut Res.

[46] Beutler E, Duron O, Kelly BM (1963). Improved method for the determination of blood glutathione. J Lab Clin Med.

[47] Cai W-q, Li S-f, Ma J-y (2004). Diseases resistance of Nile tilapia (*Oreochromis niloticus*), blue tilapia (*Oreochromis aureus*) and their hybrid (female Nile tilapia × male blue tilapia) to *Aeromonas sobria*. Aquaculture.

[48] Ellis A (1990). Lysozyme assays. J Techniques fish Immunol.

[49] Montgomery H, Dymock JJTJ. Determination of Nitrite in water. Royal Soc chemistry Thomas Graham house, science park, milton Rd, Cambridge Cb4 0wf. 1961;22:111–8.

[50] Suvarna KS, Layton C, Bancroft JD. Bancroft’s theory and practice of histological techniques. Elsevier health sciences; 2018.

[51] Bernet D, Schmidt H, Meier W, Burkhardt-Holm P, Wahli T (1999). Histopathology in fish: proposal for a protocol to assess aquatic pollution. J Fish Dis.

[52] Romano N, Zeng C (2013). Toxic effects of Ammonia, Nitrite, and nitrate to Decapod crustaceans: a review on factors influencing their toxicity, physiological consequences, and coping mechanisms. Rev Fish Sci.

[53] Shiwanand A, Tripathi G (2013). A review on ammonia toxicity in fish. Asia Pac J Life Sci.

[54] Pouder DB, Smith SA (2019). Water quality and environmental issues.

[55] Evans JJ, Pasnik DJ, Brill GC, Klesius PH (2006). Un-ionized Ammonia exposure in Nile Tilapia: toxicity, stress response, and susceptibility to *Streptococcus agalactiae*. North Am J Aquaculture.

[56] Abdel-Latif HMR, Shukry M, Abd-elaziz RA (2022). Clinico-pathological findings and expression of inflammatory cytokines, apoptosis, and oxidative stress-related genes draw mechanistic insights in Nile tilapia reared under ammonia-N exposure and *Aeromonas hydrophila* challenge. Fish Shellfish Immunol.

[57] Qin C, Zhao D, Gong Q, Qi Z, Zou Y, Yue X (2013). Effects of pathogenic bacterial challenge after acute sublethal ammonia-N exposure on heat shock protein 70 expression in Botia reevesae. Fish Shellfish Immunol.

[58] Eissa AE, Zaki MM, Aziz AA (2010). *Flavobacterium columnare*/*Myxobolus tilapiae* concurrent infection in the earthen pond reared Nile tilapia (*Oreochromis niloticus*) during the early summer. Interdisciplinary Bio Cent.

[59] Paredes-Trujillo A, Mendoza-Carranza M (2022). A systematic review and meta-analysis of the relationship between farm management, water quality and pathogen outbreaks in tilapia culture. J Fish Dis.

[60] Nofal MI, Abdel-Latif HMR (2017). Ectoparasites and bacterial co-infections causing summer mortalities among cultured fishes at Al-Manzala with special reference to water quality parameters. Life Sci J.

[61] Amal MNA, Saad MZ, Zahrah AS, Zulkafli AR (2015). Water quality influences the presence of *Streptococcus agalactiae* in cage cultured red hybrid tilapia, *Oreochromis niloticus* × *Oreochromis mossambicus*. Aquac Res.

[62] El-Greisy ZAE-B, Elgamal AEE, Ahmed NAM (2016). Effect of prolonged ammonia toxicity on fertilized eggs, hatchability and size of newly hatched larvae of Nile tilapia, *Oreochromis niloticus*. Egypt J Aquat Res.

[63] Abreu REF, Magalhães TC, Souza RC, Oliveira STL, Ibelli AMG, Demarqui FN (2018). Environmental factors on virulence of *Aeromonas hydrophila*. Aquac Int.

[64] Farmer BD, Mitchell AJ, Straus DL (2011). The effect of high total ammonia concentration on the survival of channel catfish experimentally infected with *Flavobacterium columnare*. J Aquat Anim Health.

[65] Witeska M, Kondera E, Bojarski B. Hematological and hematopoietic analysis in Fish Toxicology—A. Rev Anim [Internet]. 2023; 13(16). 10.3390/ani13162625 PMC10451336 37627416

[66] Fazio F (2019). Fish hematology analysis as an important tool of aquaculture: a review. Aquaculture.

[67] Seibel H, Baßmann B, Rebl A. Blood will tell: what hematological analyses can reveal about Fish Welfare. Front Veterinary Sci. 2021;8. 10.3389/fvets.2021.616955 PMC8042153 33860003

[68] Handayani KS, Soegianto A, Lignot J-H (2020). Change of osmoregulatory and hematological parameters in tilapia (*Oreochromis niloticus*) after exposure to sublethal mercury concentrations. Emerg Contaminants.

[69] Knoph MB, Thorud K (1996). Toxicity of ammonia to Atlantic salmon (*Salmo salar* L.) in seawater—effects on plasma osmolality, ion, ammonia, urea and glucose levels and hematologic parameters. Comp Biochem Physiol Part A: Physiol.

[70] Petitjean Q, Jean S, Gandar A, Côte J, Laffaille P, Jacquin L (2019). Stress responses in fish: from molecular to evolutionary processes. Sci Total Environ.

[71] Yi Z, Yan J, Ding Z, Xie J (2023). Purification and characterizations of a novel extracellular protease from *Shewanella putrefaciens* isolated from bigeye tuna. Food Bioscience.

[72] Wu Z-Y, Ho S-P, Cheng J-F, Tung K-C, Hong Y-K, Chen S-Y (2018). Whole-genome characterization of *Shewanella algae* strain SYT3 isolated from seawater reveals insight into hemolysis. Future Microbiol.

[73] Topić Popović N, Čižmek L, Babić S, Strunjak-Perović I, Čož-Rakovac R (2023). Fish liver damage related to the wastewater treatment plant effluents. Environ Sci Pollut Res Int.

[74] Ardeshir RA, Movahedinia A-A, Rastgar S (2017). Fish liver biomarkers for heavy metal pollution: a review article. Am J Toxicol.

[75] Sopinka NM, Donaldson MR, O’Connor CM, Suski CD, Cooke SJ. Stress indicators in fish. Fish physiology. Volume 35. Elsevier; 2016. pp. 405–62.

[76] Lemos LS, Angarica LM, Hauser-Davis RA, Quinete N. Cortisol as a stress Indicator in Fish: sampling methods, Analytical techniques, and Organic Pollutant exposure assessments. Int J Environ Res Public Health. 2023;20(13). 10.3390/ijerph20136237 PMC10341563 37444085

[77] Zeitoun MM, El-Azrak KE-DM, Zaki MA, Nemat-Allah BR, Mehana E-SE (2016). Effects of ammonia toxicity on growth performance, cortisol, glucose and hematological response of Nile Tilapia (*Oreochromis niloticus*). Aceh J Anim Sci.

[78] Dawood MAO, Sewilam H (2023). The combined effects of salinity and ammonia on the growth behavior, stress-related markers, and hepato‐renal function of common carp (*Cyprinus carpio*). J Experimental Zool Part A: Ecol Integr Physiol.

[79] Benli A, Yildiza HY. Blood parameters in Nile tilapia (*Oreochromis niloticus* L.) spontaneously infected with *Edwardsiella tarda*. Aquac Res. 2004;35(14).

[80] Baldissera MD, Souza CF, Parmeggiani B, Leipnitz G, Verdi CM, Santos RV (2018). The disturbance of antioxidant/oxidant balance in fish experimentally infected by *Aeromonas caviae*: relationship with disease pathophysiology. Microb Pathog.

[81] Bandeira Junior G, Baldisserotto B (2021). Fish infections associated with the genus Aeromonas: a review of the effects on oxidative status. J Appl Microbiol.

[82] Zhao L, Cui C, Liu Q, Sun J, He K, Adam AA (2020). Combined exposure to hypoxia and ammonia aggravated biological effects on glucose metabolism, oxidative stress, inflammation and apoptosis in largemouth bass (*Micropterus salmoides*). Aquat Toxicol.

[83] Kim J-H, Cho J-H, Kim S-R, Hur YB (2020). Toxic effects of waterborne ammonia exposure on hematological parameters, oxidative stress and stress indicators of juvenile hybrid grouper, *Epinephelus lanceolatus*♂× *Epinephelus fuscoguttatus*♀. Environ Toxicol Pharmacol.

[84] Guo M, Xu Z, Zhang H, Mei J, Xie J. The effects of Acute exposure to Ammonia on oxidative stress, Hematological Parameters, Flesh Quality, and Gill Morphological changes of the large yellow croaker (*Larimichthys crocea*). Anim [Internet]. 2023; 13(15). 10.3390/ani13152534 PMC10417668 37570342

[85] Das C, Thraya M, Vijayan MM (2018). Nongenomic cortisol signaling in fish. Gen Comp Endocrinol.

[86] Nadeem A, Al-Harbi NO, Ahmad SF, Ibrahim KE, Siddiqui N, Al-Harbi MM (2018). Glucose-6-phosphate dehydrogenase inhibition attenuates acute lung injury through reduction in NADPH oxidase-derived reactive oxygen species. Clin Exp Immunol.

[87] Silvestrini A, Meucci E, Ricerca BM, Mancini A. Total antioxidant capacity: biochemical aspects and clinical significance. Int J Mol Sci. 2023;24(13). 10.3390/ijms241310978 PMC10341416 37446156

[88] Nandi A, Yan LJ, Jana CK, Das N (2019). Role of catalase in oxidative stress- and Age-Associated Degenerative diseases. Oxid Med Cell Longev.

[89] Averill-Bates DA (2023). The antioxidant glutathione. Vitam Horm.

[90] Uribe-Querol E, Rosales C (2017). Control of phagocytosis by Microbial pathogens. Front Immunol.

[91] Schairer DO, Chouake JS, Nosanchuk JD, Friedman AJ (2012). The potential of nitric oxide releasing therapies as antimicrobial agents. Virulence.

[92] Al-Qahtani AA, Alhamlan FS, Al-Qahtani AA. Pro-inflammatory and anti-inflammatory interleukins in Infectious diseases: a Comprehensive Review. Trop Med Infect Disease. 2024;9(1). 10.3390/tropicalmed9010013 PMC10818686 38251210

[93] Urbinati EC, Zanuzzo FS, Biller JD. Chapter 5 - stress and immune system in fish. In: Baldisserotto B, Urbinati EC, Cyrino JEP, editors. Biology and Physiology of Freshwater Neotropical Fish. Academic; 2020. pp. 93–114.

[94] Esteban MA, Rodrıguez A, Ayala AG, Meseguer J (2004). Effects of high doses of cortisol on innate cellular immune response of seabream (*Sparus aurata* L). Gen Comp Endocrinol.

[95] Fast MD, Hosoya S, Johnson SC, Afonso LO (2008). Cortisol response and immune-related effects of Atlantic salmon (*Salmo salar* Linnaeus) subjected to short- and long-term stress. Fish Shellfish Immunol.

[96] Dai C, Zheng J, Qi L, Deng P, Wu M, Li L et al. Chronic stress boosts systemic inflammation and compromises antiviral innate immunity in *Carassius gibel*. Front Immunol. 2023;14. 10.3389/fimmu.2023.1105156 PMC9939519 36814911

[97] Yada T, Tort L. 10 - stress and Disease Resistance: Immune System and Immunoendocrine interactions. In: Schreck CB, Tort L, Farrell AP, Brauner CJ, editors. Fish Physiology. Volume 35. Academic; 2016. pp. 365–403.

[98] Esam F, Khalafalla MM, Gewaily MS, Abdo S, Hassan AM, Dawood MAO (2022). Acute ammonia exposure combined with heat stress impaired the histological features of gills and liver tissues and the expression responses of immune and antioxidative related genes in Nile tilapia. Ecotoxicol Environ Saf.

[99] Benli AC, Köksal G, Ozkul A (2008). Sublethal ammonia exposure of Nile tilapia (Oreochromis niloticus L.): effects on gill, liver and kidney histology. Chemosphere.

